# Proteolytic Processing of Plant Proteins by Potyvirus NIa Proteases

**DOI:** 10.1128/JVI.01444-21

**Published:** 2022-01-26

**Authors:** Huogen Xiao, Etienne Lord, Hélène Sanfaçon

**Affiliations:** a Summerland Research and Development Centre, Agriculture and Agri-Food Canada, Summerland, British Columbia, Canada; University of Maryland, College Park

**Keywords:** plant viruses, plant-virus interaction, plum pox virus, potyvirus, proteases

## Abstract

The NIa protease of potyviruses is a chymotrypsin-like cysteine protease related to the picornavirus 3C protease. It is also a multifunctional protein known to play multiple roles during virus infection. Picornavirus 3C proteases cleave hundreds of host proteins to facilitate virus infection. However, whether or not potyvirus NIa proteases cleave plant proteins has so far not been tested. Regular expression search using the cleavage site consensus sequence [EQN]xVxH[QE]/[SGTA] for the plum pox virus (PPV) protease identified 90 to 94 putative cleavage events in the proteomes of Prunus persica (a crop severely affected by PPV), Arabidopsis thaliana, and Nicotiana benthamiana (two experimental hosts). *In vitro* processing assays confirmed cleavage of six A. thaliana and five P. persica proteins by the PPV protease. These proteins were also cleaved *in vitro* by the protease of turnip mosaic virus (TuMV), which has a similar specificity. We confirmed *in vivo* cleavage of a transiently expressed tagged version of AtEML2, an EMSY-like protein belonging to a family of nuclear histone readers known to be involved in pathogen resistance. Cleavage of AtEML2 was efficient and was observed in plants that coexpressed the PPV or TuMV NIa proteases or in plants that were infected with TuMV. We also showed partial *in vivo* cleavage of AtDUF707, a membrane protein annotated as lysine ketoglutarate reductase *trans*-splicing protein. Although cleavage of the corresponding endogenous plant proteins remains to be confirmed, the results show that a plant virus protease can cleave host proteins during virus infection and highlight a new layer of plant-virus interactions.

**IMPORTANCE** Viruses are highly adaptive and use multiple molecular mechanisms to highjack or modify the cellular resources to their advantage. They must also counteract or evade host defense responses. One well-characterized mechanism used by vertebrate viruses is the proteolytic cleavage of host proteins to inhibit the activities of these proteins and/or to produce cleaved protein fragments that are beneficial to the virus infection cycle. Even though almost half of the known plant viruses encode at least one protease, it was not known whether plant viruses employ this strategy. Using an *in silico* prediction approach and the well-characterized specificity of potyvirus NIa proteases, we were able to identify hundreds of putative cleavage sites in plant proteins, several of which were validated by downstream experiments. It can be anticipated that many other plant virus proteases also cleave host proteins and that the identification of these cleavage events will lead to novel antiviral strategies.

## INTRODUCTION

Plant viruses have a long coevolutionary history with their hosts (1, 2). This has driven the fine-tuning of highly adapted multitasking viral proteins that modify the host cellular metabolism and/or counteract host defense responses to create an optimized environment for virus infection (3–5). Viral proteases are prime examples of this multifunctionality. While their primary role is to orchestrate the regulated cleavage of viral polyproteins at specific sites, many plant virus proteases also have other functions, e.g., suppression of antiviral RNA silencing and/or other plant defense responses, viral systemic movement, viral RNA replication, host adaptation, and vector transmission (6, 7). In some cases, these additional functions do not depend on the protease catalytic activity. Rather, they are driven by protein domains that are distinct from the protease proteolytic domain. The potyvirus HC-Pro protein is a well-characterized example of such functional domain separation and includes an N-terminal aphid transmission domain, a central silencing suppression domain, and a C-terminal protease domain (8). However, it is also possible that the virulence function associated with some plant virus proteases depends on the cleavage of host proteins to modify the cellular environment, as shown for vertebrate virus proteases (9–14). Cellular targets of plant virus proteases have not yet been identified.

The translation initiation factor eIF4G, which has been shown to be cleaved by the poliovirus 2A protease (15) and by the foot and mouth disease virus (FMDV) leader protease (16), was the first identified cellular target of viral proteases. Since then, the list of cellular proteins cleaved by vertebrate virus proteases has been steadily growing and includes transcription and translation factors, components of the innate immune response, nuclear import factors, and metabolic enzymes (9–14). Recent proteomic studies identified hundreds of new host proteins that are cleaved by various enterovirus 3C proteases (17, 18). In many cases, the main impact of the proteolytic cleavage of host proteins is the inactivation of their function. Recently characterized examples include the cleavage of the signaling proteins TBK1 and MAVS by the FMDV leader protease that prevents the transcriptional activation of interferon α/β (19) and the cleavage of the 4-dehydrocholesterol reductase by the hepatitis C virus NS3-4 protease that reduces the conversion of desmosterol to cholesterol, changes the lipid environment of the cell, and facilitates viral RNA replication in the membrane-associated virus factories (20). However, host protein fragments produced by viral proteases can also retain functions that are advantageous for virus infection. For example, the C-terminal eIF4G fragment produced after cleavage by picornavirus proteases lacks the eIF4E-binding domain required for cap-dependent cellular mRNA translation, but is fully functional for the cap-independent translation of picornavirus RNAs (21, 22). In addition, cleaved fragments could act as *trans*-dominant negative mutants, as shown for the C-terminal fragment of Ras-GAP SH3 domain binding protein-1 (G3BP1), which is released after cleavage by the coxsackievirus type B3 3C protease and inhibits stress granule formation to enhance virus accumulation (23).

**FIG 1:**
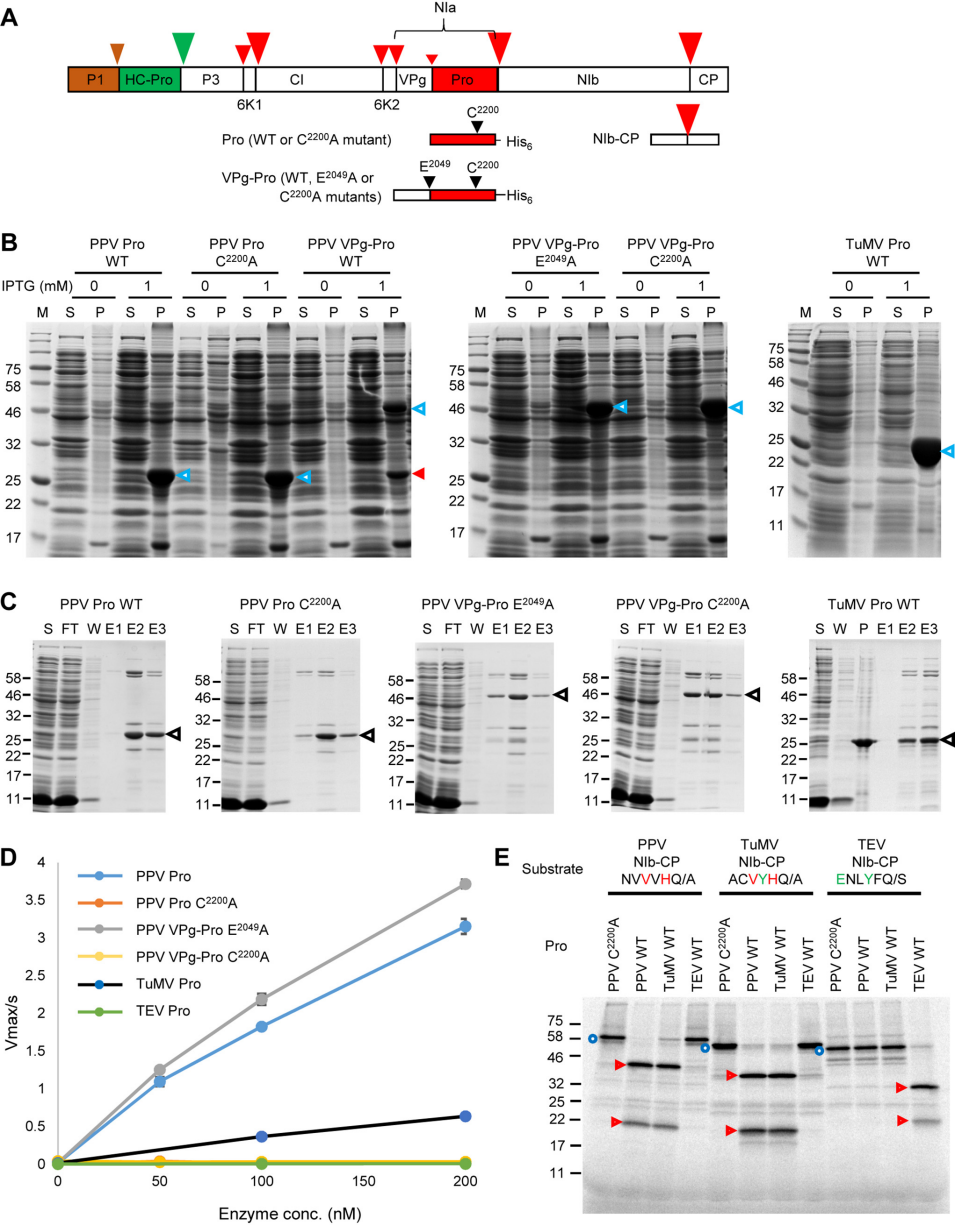
Activity and specificity of purified recombinant potyvirus proteases. (A) Schematic representation of the PPV polyprotein. Autoproteolytic cleavage sites that are processed by the P1 and HC-Pro proteases are indicated with brown and green arrowheads, respectively. Cleavage sites that are processed by the NIa protease are marked with red arrowheads. The size of the arrowhead represents the relative cleavage efficiency at each site. Portions of the polyprotein used in this study are shown, including the Pro and VPg-Pro used for production of recombinant proteases and the NIb-CP partial polyprotein used as a substrate for the proteases. The position of the catalytic triad cysteine (C^2200^, numbering from the beginning of the PPV polyprotein) and of the glutamic acid (E^2049^) at the P1 position of the VPg-Pro cleavage site are also shown. (B) Expression of various forms of the PPV and TuMV NIa proteases. Protein expression was induced by the addition of 1 mM IPTG, and protein extracts were run on SDS-PAGE after separation into supernatant fractions (S) containing soluble proteins and pellet fractions (P) containing insoluble proteins. Control lanes (0 mM IPTG) were included that corresponded to S and P fractions from Escherichia coli grown in the absence of IPTG. Migration of the full-length expressed proteins is shown with the blue arrowheads. The mature Pro released after autocatalytic cleavage of the PPV VPg-Pro WT is indicated by the red arrowhead. Please note that this fragment comigrates with the mature Pro expressed from the PPV Pro wild type (WT) and PPV Pro C^2200^A constructs (calculated molecular mass, 28.9 kDa). M, protein ladder with the molecular mass (kDa) indicated on the left. (C) Purification of PPV and TuMV proteases. SDS-PAGE gels showing the purification of recombinant proteases. Proteases were partially purified from the soluble protein fraction using the Ni-NTA resin column. Expected migration of the expressed proteases is indicated by the black arrowheads. Other bands are likely minor contaminant E. coli proteins. FT, flowthrough from the Ni-NTA resin column; W, wash collections; E, elution fractions; S, supernatant fraction containing soluble proteins; P, pellet fraction containing insoluble proteins. The migration of molecular mass markers is indicated on the left. (D) Activity of purified potyvirus proteases tested using the MCA-QA-DNP fluorescent peptide substrate, which is derived from the PPV NIb-CP cleavage site. Reactions were conducted in the assay buffer (50 mM Tris-HCl [pH 7.5], 150 mM NaCl, 1 mM EDTA, 1 mM DTT, and 0.1% Brij35). The substrate concentration was 5 μM, and the enzyme concentration ranged from 50 to 200 nM. Values are reported as the mean ± standard deviation (SD) from triplicate measurements. (E) *In vitro* processing of partial viral polyproteins (PPV, TuMV, or TEV NIb-CP) by the PPV, TuMV, and TEV Pro[s]. NIb-CP from PPV, TuMV, and TEV were synthesized *in vitro* using rabbit reticulocytes extracts. Translation products were then incubated at 16°C for 16 h in the presence of 50 nM wild-type or mutated PPV, TuMV, or TEV protease (as indicated above each lane). The full-length precursor protein and cleavage products were separated by 12% SDS-PAGE. The migration positions of molecular mass markers are indicated on the left. Full-length substrate proteins are indicated with blue circles, and cleaved fragments are indicated with red arrowheads.

The genus Potyvirus (family Potyviridae), which includes more than 150 species, is an ancient lineage of economically important plant positive-strand RNA viruses (24, 25). Notable potyviruses include plum pox virus (PPV), which causes sharka disease in stone fruit trees (26, 27), and turnip mosaic virus (TuMV), an important pathogen of Brassica species (28). Potyviruses encode a large polyprotein, which is cleaved by three viral proteases (25, 26, 29, 30) (Fig. 1A). The N-terminal P1 and HC-Pro proteases each cleave the polyprotein at a single site to allow their autocatalytic release. Because these proteases only function in *cis* (31–33), they are unlikely to process host proteins, which would require a *trans*-cleavage event. The NIa protease is the main potyvirus protease, responsible for the *cis-* and *trans-*processing of the polyprotein at seven sites. Because of its stringent specificity and efficient *trans-*cleavage activity, the well-characterized NIa protease from tobacco etch virus (TEV) is frequently used for biotechnological applications, notably for the release of expressed recombinant proteins from various tags (34). Potyvirus NIa proteases are closely related to the picornavirus 3C proteases, which are known to target host proteins. Like the 3C proteases, NIa proteases adopt a fold similar to that of the cellular chymotrypsin serine protease, but they have a cysteine instead of a serine as the nucleophile (6). The 3C and 3C-like proteases are encoded by large groups of viruses that infect vertebrates, invertebrates, plants, fungi, and lower eukaryotes, indicating that these protease domains were acquired early in the evolutionary history of these viruses and have coadapted with the host cellular environment (2, 6).

In infected cells, the NIa protease is detected in various forms (25, 29). The stable intermediate NIa polyprotein is the predominant form. It contains the domains for the genome-linked viral protein (VPg) and the protease (Pro) and accumulates as inclusion bodies in the nucleus, although it is also detected in the cytoplasm (35). Processing of this VPg-Pro polyprotein at a suboptimal internal cleavage site regulates the slow release of the mature VPg and Pro, both of which also accumulate predominantly in the nucleus (35, 36). The 6K2-VPg-Pro intermediate polyprotein is detected in association with endoplasmic reticulum (ER)-derived membranous vesicles active in viral replication (37–40). In addition, it was shown previously that the TuMV 6K2-VPg-Pro polyprotein ultimately accumulates in chloroplast-associated replication complexes that develop by fusion of ER membranes with chloroplasts (41, 42). Although all forms of the NIa protease have proteolytic activity, the presence of the VPg domain on the VPg-Pro polyprotein was shown to enhance the protease activity (43).

Like many other viral proteases, the NIa protease is multifunctional. It interacts with the viral RNA-dependent RNA polymerase and with the viral RNA and stimulates viral RNA replication (44–46). The pepper mottle virus NIa protease is a pathogenicity factor, and this function has been attributed at least in part to its ability to inhibit transcriptional gene silencing and reverse global methylation of the plant genome (47). The NIa proteases of two potyviruses play a role in superinfection exclusion (48), and the papaya ringspot virus NIa protease determines host specificity (49). The potato virus Y NIa protease is the elicitor of the *Ry* resistance gene in potato, and the proteolytic activity of the protease was shown to be required for this function (50). It was suggested that cleavage of a host protein by the viral protease may contribute to the elicitation of the defense response (50). Finally, the TuMV NIa protease stimulates reproduction of the aphid vector by disrupting the plant ethylene defense response (51, 52). The protease is relocalized to the vacuole in aphid-infected plants, and this relocalization is necessary for the enhancement of aphid performance (53). The NIa intermediate polyprotein interacts with many host proteins, through either the VPg or the protease domains (54, 55). While the molecular mechanisms enabling the manipulation of multiple layers of the plant-virus and plant-vector interfaces by the NIa protease are not yet completely deciphered, it is clear that the NIa protease plays a central role in the virus infection process.

Here, we investigated whether or not the NIa protease cleaves host proteins during virus infection. To identify possible host protein targets of the PPV NIa protease, we took advantage of its well-characterized specificity (56–61). We searched the plant proteome for the consensus cleavage site sequence [EQN]xVxH[QE]/[STGA], where amino acid residues in square brackets are interchangeable, x indicates any residues, and the cleavage site is indicated by the oblique bar. We identified putative cleavage sites in 91 Arabidopsis thaliana proteins, 90 Prunus persica proteins, and 94 Nicotiana benthamiana proteins. Using *in vitro* processing assays, we confirmed cleavage of six *A. thaliana* proteins and five peach proteins by the PPV and TuMV NIa proteases, which have similar cleavage site specificities (62, 63). We also confirmed *in vivo* cleavage of two transiently expressed tagged host proteins in plants that were also expressing the PPV or TuMV NIa proteases or that were infected by TuMV.

## RESULTS

### Cleavage site consensus sequences for the PPV, TuMV, and TEV NIa proteases.

The cleavage site specificities of the PPV, TuMV, and TEV NIa proteases are well characterized (30). The consensus sequence for viral polyprotein cleavage sites recognized by the PPV NIa protease is [EQN]xVxH[QE]/[STGA] (56–61), with the P1, P2, and P4 positions being the most conserved. The specificity of the TuMV NIa protease is similar with the consensus cleavage site sequence xxVxH[QE]/[AST] that includes the same preferred amino acids at the P1, P2, and P4 positions (62, 64). The TEV NIa protease has a distinct specificity with the consensus cleavage site sequence Ex[VIL]Yx[QE]/[SG], where the P6, P3, and P1 positions are the most conserved (65–67). We reexamined these specificities by comparing the amino acid sequences at the P6 to P1′ positions of the seven cleavage sites present on the polyprotein using all full genome sequences currently available in the NCBI database (Table 1; see also Tables S1 to S3 in the supplemental material). This analysis confirmed the cleavage site consensus sequences, although it also highlighted the occasional presence of divergent amino acids at key positions. The P1 position showed the expected strong preference for Q or E, but alternative amino acids were occasionally found (D or P for PPV and D or R for TuMV), suggesting some unexpected flexibility at that position. At the P2 position, H was preferred for PPV and TuMV, but other amino acids were also found (predominantly N or T for PPV and A for TuMV). In the case of TEV, a slight preference for F was noted at that position. The P3 position did indeed show a strong preference for Y in TEV cleavage sites, but other amino acids were also present in some cleavage sites. In PPV and TuMV cleavage sites, V, H, Y, and D were most common at that position, but other amino acids were also found. The P4 position was the most conserved in PPV and TuMV cleavage sites, with a clear preference for V. Only a few cleavage sites showed a deviation from this consensus with A at that position. In contrast, L, V, or I was found in the P4 position of TEV cleavage sites. The P6 position showed a strict requirement for E in TEV cleavage sites, a preference for E, Q, or N in PPV cleavage sites, and a preference for E, V, T, P, and K in TuMV cleavage sites. However, a diverse range of other amino acids were also present at the P6 position of both PPV and TuMV cleavage sites. At the P1′ position, G, S, and A were commonly found for all three viruses; T was also commonly found in PPV and TuMV cleavage sites and N in TuMV cleavage sites. Other amino acids (V or M) were only occasionally seen at this position for PPV and TuMV cleavage sites.

**TABLE 1:**
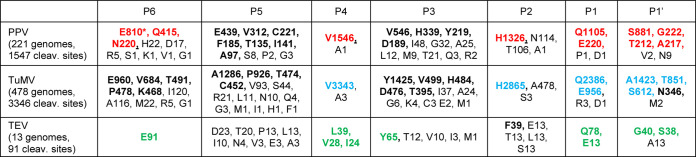
Cleavage site sequences for three potyvirus NIa proteases^*a*^

a *, Amino acids present at each position are shown by single-letter code. The number after each amino acid represents the total number of cleavage sites that contain this amino acid at this position. Red letters indicate the previously identified PPV cleavage site consensus sequence that was used to search plant proteomes. Blue and green letters represent the previously identified consensus sequences for TuMV and TEV cleavage sites, respectively. Please refer to Tables S1 to S3 in the supplemental material for detailed information for each individual cleavage sites.

### Purification of recombinant potyvirus NIa proteases and confirmation of their *in vitro* activity and specificity.

The TEV mature NIa protease (Pro) is available commercially. We purified the PPV mature Pro and intermediate polyprotein VPg-Pro, as well as the TuMV mature Pro, after expression in Escherichia coli (Fig. 1A to C). To prevent the release of Pro from the VPg-Pro intermediate by autocatalytic proteolytic cleavage at the VPg-Pro cleavage site, we introduced a mutation at the P1 position of this cleavage site (mutation E^2049^A) (Fig. 1A). To ensure that any detected proteolytic processing events were specific to the NIa protease activity, we also produced inactive versions of Pro and VPg-Pro, which included a mutation of the catalytic cysteine (mutation C^2200^A) (Fig. 1A). All proteases were successfully expressed in E. coli under standard growing conditions (30°C and 1 mM isopropyl-β-d-thiogalactopyranoside [IPTG] for induction), although the proteases were mostly found in the insoluble fraction (Fig. 1B). The wild-type (WT) PPV VPg-Pro (51.5 kDa) was partially cleaved to allow the release of the mature Pro (28.9 kDa) (Fig. 1B). Mutation of the VPg-Pro cleavage site (PPV VPg-Pro E^2049^A) or of the protease catalytic residue (PPV VPg-Pro C^2200^A) prevented the autocleavage of VPg-Pro (Fig. 1B). After optimization, soluble PPV Pro WT, PPV Pro C^2200^A, PPV VPg-Pro E^2049^A, PPV VPg-Pro C^2200^A, and TuMV Pro were partially purified by Ni^2+^ affinity chromatography (Fig. 1C). Assay conditions were optimized using the partially purified PPV Pro WT and the fluorescent peptide substrate (MCA-QA-DNP; see Materials and Methods), which incorporates the PPV NIb-CP cleavage site. The optimized activity buffer was used thereafter (see Materials and Methods).

PPV Pro, PPV VPg-Pro-E^2049^A, and TuMV Pro all cleaved the MCA-QA-DNP peptide, as demonstrated by the linear increase of fluorescence with increased concentrations of the proteases (Fig. 1D). In contrast, the active-site mutants PPV Pro-C^2200^A and PPV VPg-Pro-C^2200^A did not cleave the peptide, confirming that the proteolytic activity was not due to an E. coli contaminant present in the partially purified proteases. The *K_m_* values of PPV Pro and PPV VPg-Pro-E^2049^A were estimated to be 20.6 μM and 15.6 μM, respectively. Their *k*_cat_ values were 5.6 × 10^−2^ · sec^−1^ and 4.4 × 10^−2^ · sec^−1^, respectively. This suggests that the PPV Pro and PPV VPg-Pro-E^2049^A have comparable activities using peptide MCA-QA-DNP. TuMV Pro cleaved the MCA-QA-DNP peptide with *K_m_* and *k_cat_* values of 122.7 μM and 4.9 × 10^−2^ · sec^−1^, respectively, indicating that TuMV Pro had a lower affinity for the peptide than that of PPV Pro, although it had a similar turnover number (*k_cat_*). TEV Pro did not cleave the peptide. This was expected, given the known difference in the specificity of this protease, which requires an E at the P6 position of the cleavage site and a Y at P3, both of which are absent from the peptide sequence.

To further confirm the activity and specificity of the three proteases, we used *in vitro* processing assays to test the cleavage of partial viral polyproteins that incorporated the NIb-CP cleavage site. As expected, given their similar cleavage site specificity, the PPV and TuMV proteases were able to process the PPV and TuMV NIb-CP precursors (Fig. 1E; see Table 2 for expected molecular masses of precursor proteins and cleaved fragments). On the other hand, these proteases did not cleave the TEV NIb-CP precursor, which includes a cleavage site that lacks the conserved H at the P2 position and V at the P4 position (Fig. 1E). TEV Pro cleaved the TEV NIb-CP precursor but did not cleave the PPV NIb-CP and TuMV NIb-CP precursors, which lack the conserved Y at the P3 position and/or E at the P6 position of the cleavage sites (Fig. 1E). These results confirmed the specificity of the *in vitro* processing assay for testing proteolytic cleavage by the three NIa proteases.

**TABLE 2:**
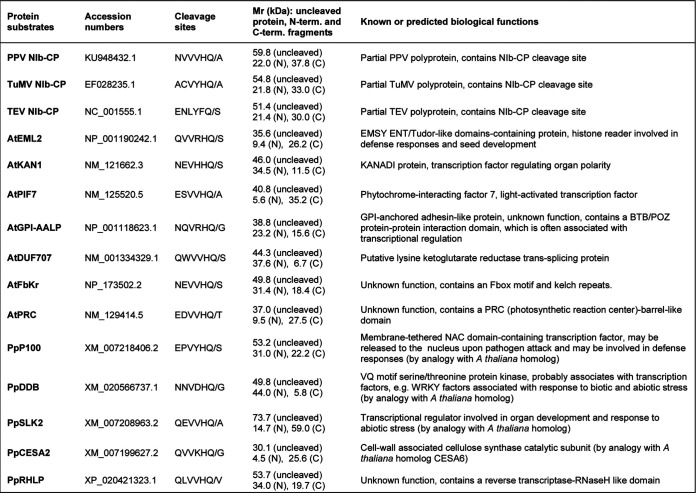
Protein substrates tested for *in vitro* cleavage by potyvirus proteases^*a*^

a NIb, nuclear inclusion protein b (RNA-dependent RNA polymerase); CP, capsid protein; At, Arabidopsis thaliana; Pp, *Prunus persica*.

### Identification of putative PPV NIa protease cleavage sites in plant proteins.

Using a regular expression search with the search term corresponding to the confirmed PPV cleavage site consensus sequence [EQN]xVxH[QE]/[STGA], we identified putative cleavage sites in 91 proteins from the *A. thaliana* proteome, 90 proteins from the P. persica proteome, and 94 proteins from the N. benthamiana proteome (see Excel Sheet S1 in the supplemental material). These numbers include protein isoforms that are produced by splicing variants (e.g., for *A. thaliana*, the 91 listed proteins were encoded by 58 individual genes). *P. persica* is an economically important horticultural host of PPV; *A. thaliana* and N. benthamiana are model experimental hosts for PPV, TuMV, and TEV (25, 26, 68).

**FIG 2:**
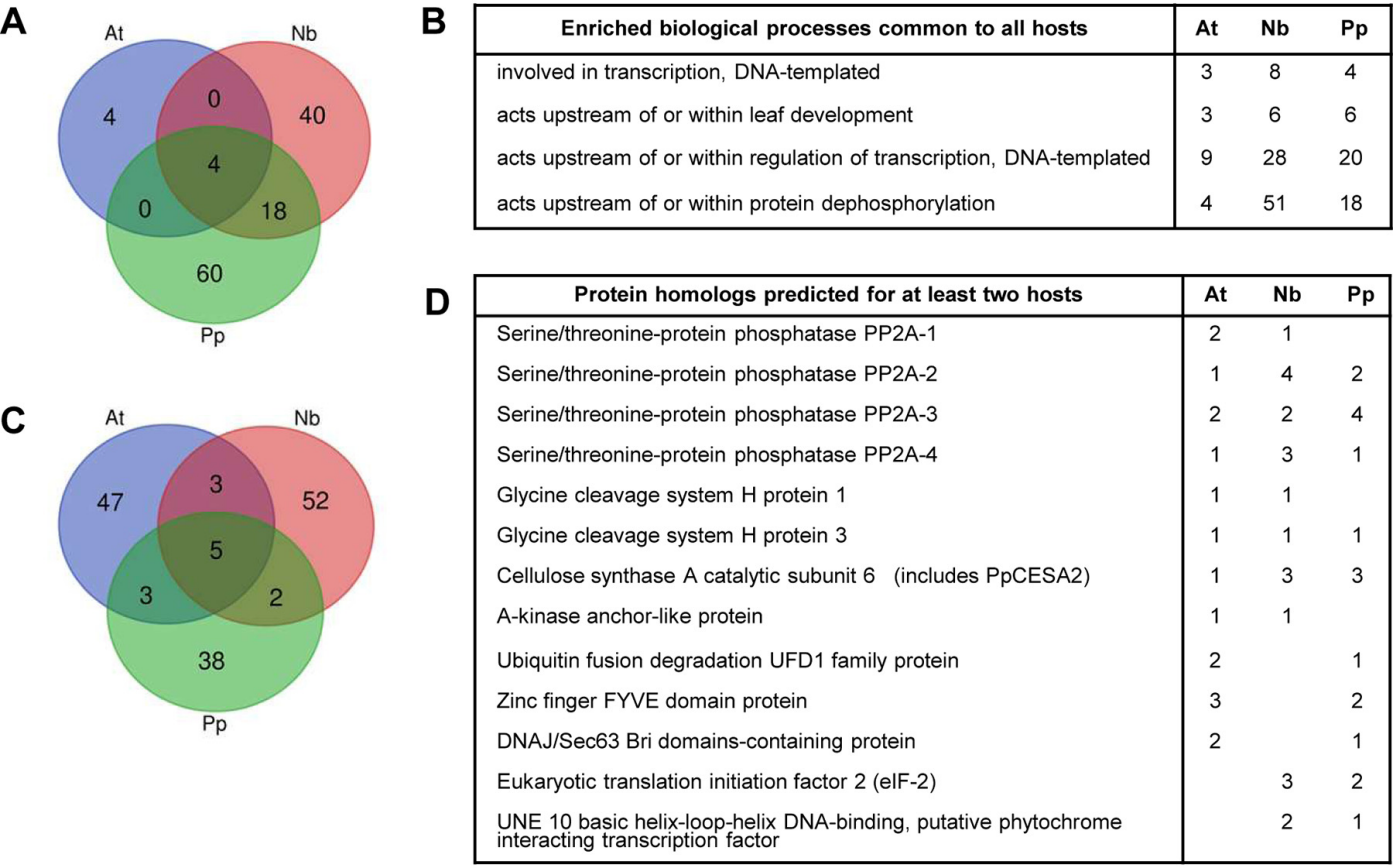
Analysis of putative protein targets in natural or experimental hosts of PPV. (A) Venn diagram showing shared enriched biological processes associated with putative target proteins in *A. thaliana* (At), N. benthamiana (Nb), and *P. persica* (Pp). Gene ontologies (GOs) associated with identified protein targets were determined using the TAIR database annotation for the closest homolog as determined by BLASTP. For each host, GOs with adjusted *P* values over 0.05, as well as GOs corresponding to fewer than three proteins, were eliminated prior to the comparison. See Excel Sheet S1 in the supplemental material for additional information. (B) List of enriched GOs (biological process) shared in all three plant hosts. The number of proteins associated with each GO is indicated for each plant host. (C) Venn diagram showing common putative protein targets among the three plant hosts. For N. benthamiana and *P. persica*, BLASTP was used to identify the most related *A. thaliana* gene. The lists of corresponding *A. thaliana* genes generated for each host were then compared. See Excel Sheet S1 for additional information. (D) List of common target proteins. The numbers of protein isoforms identified for each common gene and for each host are indicated.

The putative plant protein targets identified included transcription factors or regulators, metabolic enzymes, kinases, phosphatases, components of the plant response to pathogens or abiotic stresses, component of the intracellular protein transport network, chaperones, components of the proteasome, and ribosomal proteins, as well as many proteins of unknown function but with predicted protein-protein or protein-nucleic acid interaction domains (Table 3 and Excel Sheet S1). Gene ontology (GO) annotations revealed putative plant protein targets of the PPV NIa protease predicted to be located in the nucleus, the cytoplasm, and various organelles or intracellular membranes (Table 3 and Excel Sheet S1). GO enrichment analysis of putative target proteins from the three plant hosts revealed some commonalities, in particular an enrichment of GOs associated with transcription regulation and protein dephosphorylation (Fig. 2A and B and Excel Sheet S1).

**TABLE 3:**
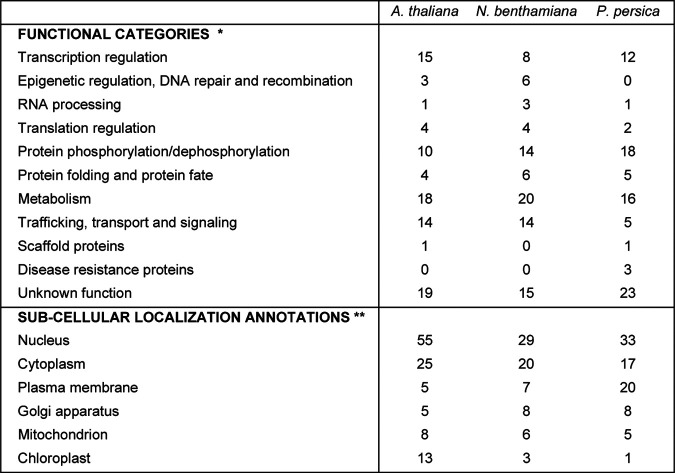
Summary of plant proteins with predicted cleavage sites^*a*^

a *, Functional categories were determined based on gene ontology (GO) annotations, BLAST searches, and/or literature searches. Please see Excel Sheet S1 for detailed information. **, Subcellular localizations were obtained from TAIR for *A. thaliana*; for N. benthamiana and *P. persica*, protein annotations of *A. thaliana* homologs identified by BLASTP were used. Only a subset of subcellular annotations that were more frequently found in the data set is shown. Please note that not all subcellular localizations have been confirmed experimentally. Please also note that many proteins have multiple subcellular localizations.

While the lists of protein targets were unique for each plant proteome tested, there were overlaps (Fig. 2C and D and Excel Sheet S1). For example, putative cleavage sites were identified in cellulose synthase catalytic subunits, serine/threonine protein phosphatase PP2A catalytic subunits, and glycine cleavage system H proteins for all three plant proteomes (Fig. 2D and Excel Sheet S1).

### *In vitro* cleavage of candidate *A. thaliana* and peach proteins by purified recombinant PPV NIa protease.

**FIG 3:**
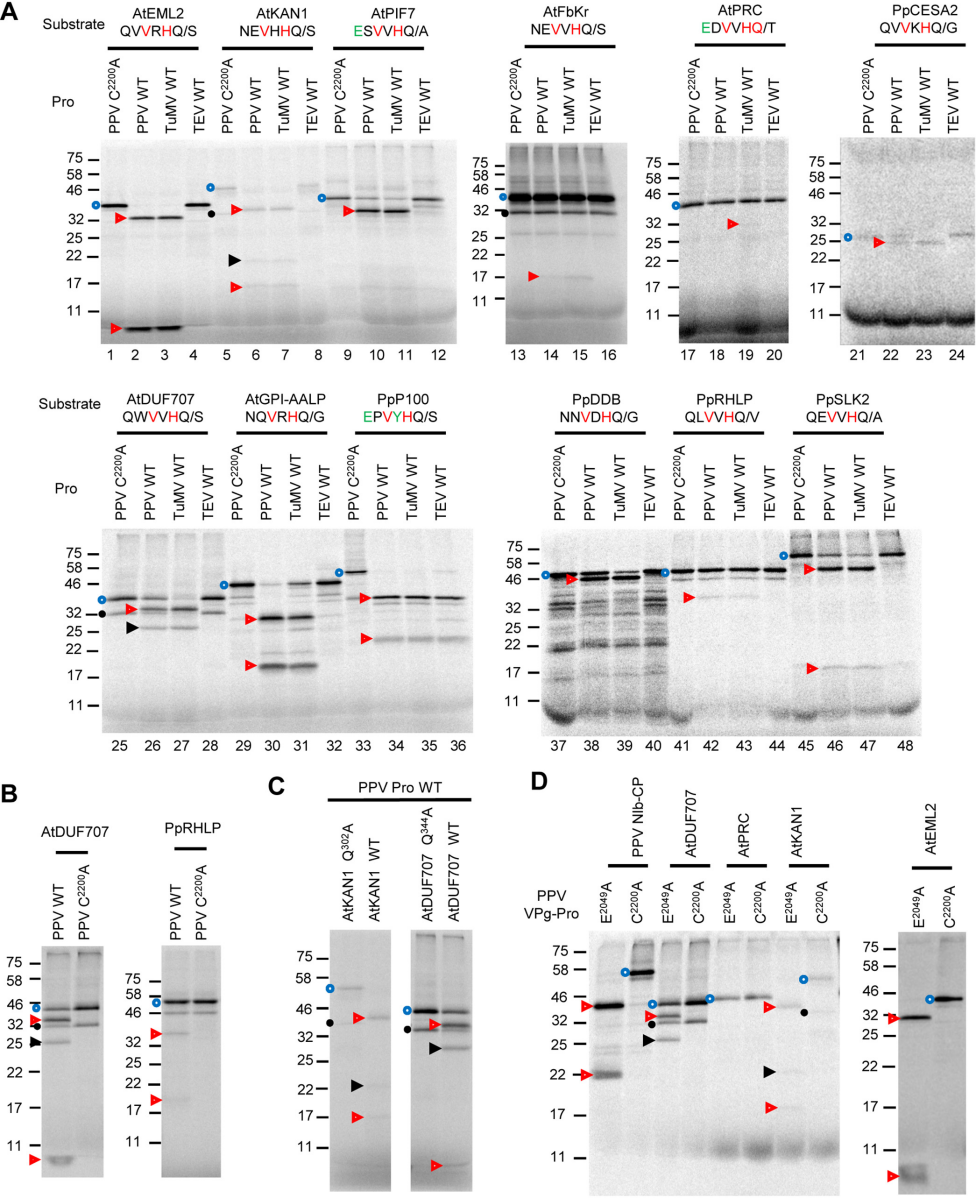
*In vitro* processing of selected *A. thaliana* and peach proteins by potyvirus NIa proteases. (A) *In vitro* processing assays in the presence of PPV, TuMV, or TEV proteases. Plant proteins were synthesized *in vitro* using rabbit reticulocyte extracts. Translation products were then incubated at 16°C for 16 h in the presence of 50 nM PPV Pro (PPV WT), PPV Pro C^2200^A (catalytically inactive mutant used as a control, PPV C^2200^A), TuMV Pro (TuMV WT), or TEV Pro (TEV WT). The amino acid sequence of the predicted cleavage site (P6 to P1′ positions) is shown. The V and H at the P4 and P2 positions, respectively, which are preferred by the PPV and TuMV Pro[s], are highlighted in red. The E and Y at the P6 and P3 positions, respectively, which are preferred by the TEV Pro, are highlighted in green. (B) Detection of the small cleaved fragment of AtDUF707 and PpRHLP after cleavage with PPV Pro WT. (C) Mutation of the amino acid at the P1 position of the predicted cleavage site prevents cleavage of AtKAN1 and AtDUF707. Wild-type or cleavage site mutant of AtKAN1 and AtDUF707 were synthesized *in vitro* and incubated with wild-type PPV Pro as described in panel A. In the mutants, the Q at the P1 position of the predicted cleavage sites was replaced by A (AtKAN1 Q^302^A and AtDUF707 Q^344^A). (D) *In vitro* processing of selected *A. thaliana* proteins by PPV VPg-Pro. Reactions were performed as for those in panel A, with the exception that the translation products were incubated with 50 nM PPV VPg-Pro E^2049^A (wild-type protease with a mutation of the VPg-Pro cleavage site to prevent release of the mature Pro) or of the catalytically inactive mutant, VPg-Pro C^2200^A. For all panels, the precursor protein and cleavage products were separated by SDS-PAGE (12% polyacrylamide for panels A and B and 15% for panel C). The migration positions of molecular mass markers (in kDa) are indicated on the left of each gel. Full-length proteins and cleaved fragments are labeled with blue circles and red arrowheads, respectively. In some cases, secondary bands present in the uncleaved translation products (labeled with black circles) are likely produced by internal translation initiation, and the cleaved fragments derived from these bands are labeled with black arrowheads.

We selected the following seven *A. thaliana* proteins: AtEML2 (EMSY N terminus [ENT]/Agenet-Tudor-like domains-containing protein, a putative histone reader), AtKAN1 (KANADI protein 1, a putative transcription factor), AtPIF7 (phytochrome-interacting factor 7), AtGPI-AALP (GPI-anchored adhesin-like protein), AtDUF707 (lysine ketoglutarate reductase *trans*-splicing protein), AtFbKr (F-box/kelch repeat-like protein), and AtPRC (PRC-barrel-like protein), which have predicted cleavage sites that are well aligned with the PPV cleavage site consensus sequence, all, including a Q at the P1 position (Table 2). The first five proteins were readily cleaved by the wild-type PPV Pro (WT), but not by the catalytically inactive mutant (C^2200^A), and produced cleaved fragments of the expected molecular masses (Fig. 3A; see Table 2 for calculated molecular masses of full-length proteins and cleaved fragments). The full-length AtEML2, AtKAN1, and AtGPI-AALP proteins (Fig. 3A, lanes 1, 5, and 29, blue circles) were completely cleaved into two fragments of the expected size (Fig. 3A, lanes 2, 6, and 30, red arrowheads). In the AtKAN1 translation products incubated with the mutated protease, a secondary band (Fig. 3A, lane 5, black circle) is likely produced by translation initiation at an internal AUG. This band is apparently also cleaved by the PPV Pro (cleaved fragment indicated with a black arrowhead in Fig. 3A, lane 6). For AtPIF7, the expected sizes of the fragments after cleavage by the PPV Pro are 35.2 and 5.6 kDa. We detected the 35.2-kDa fragment after incubation with the WT PPV Pro, but the 5.6 kDa fragment was not detectable (Fig. 3A, lane 10). Because there are only one cysteine and one methionine residue in the predicted 5.6-kDa fragment, the radioactive signal may not have been strong enough to produce a noticeable band. The full-length AtDUF707 protein was partially cleaved, and the two expected cleaved fragments were detected (Fig. 3A, lane 26, and Fig. 3B). As observed for AtPIF7, a secondary band that was likely produced by internal translation initiation (Fig. 3A, lane 25, black circle) was also apparently cleaved by PPV Pro (Fig. 3A, lane 26, black arrowhead). We did not observe cleavage of AtPRC by PPV Pro (Fig. 3A, lane 18). Cleavage of AtFbKr was observed but was inefficient (Fig. 3A, lane 14). The full-length AtFbKr protein contains 39 methionines and cysteines and was very efficiently radiolabeled. The predicted C-terminal 18.4-kDa fragment contains 31 methionines and cysteines, but it was only weakly detected. The 31.4-kDa N-terminal fragment that contains 8 methionines and cysteines was not clearly detected, although it may also have comigrated with a secondary band migrating at that position (Fig. 3A, lanes 13 and 14, black circle).

We introduced mutations at the P1 position of the predicted cleavage sites of AtKAN1 and AtDUF707, replacing the Q with A (mutants AtKAN1 Q^302^A and AtDUF707 Q^344^A). Cleavage was not observed for these mutants, strongly suggesting that the cleavage occurred at the predicted cleavage sites (Fig. 3C).

We also tested the following five candidate proteins from peach: NAC domain-containing protein 100 (PpP100), probable serine/threonine-protein kinase DDB (PpDDB), probable transcriptional regulator SLK2 (PpSLK2), cellulose synthase A catalytic subunit 2 (PpCESA2), and RNase H-like protein (PpRHLP) (Table 2). All five candidate proteins were cleaved by PPV Pro, although at various efficiencies. PpP100 and PpSLK2 were readily cleaved by PPV Pro, and the two expected fragments were detected (Fig. 3A, lanes 34 and 46). PpDDB was also cleaved by PPV Pro, allowing the detection of the expected 44-kDa cleaved fragment (Fig. 3A, lane 38). The truncated PpCESA2 protein contains only 7 cysteines and methionines and was inefficiently labeled. However, cleavage by PPV Pro did occur, and the expected 25.6-kDa fragment was observed (Fig. 3A, lane 22). PpRHLP was only partially processed to produce the two expected fragments (Fig. 3A, lane 42, and Fig. 3B). The putative cleavage site in this protein was identified using a more degenerate search and slightly differed from the PPV protease consensus in that it included a V at the P1′ position (Table 2). Although this amino acid has been found in two PPV cleavage sites (Table 1), it is not commonly occurring at the P1 position of PPV polyprotein cleavage sites.

In virus-infected cells, only a small percentage of the VPg-Pro precursor is processed to release the mature Pro (35). Next, we tested the cleavage of host proteins by the PPV VPg-Pro *in vitro*. The results confirmed cleavage of AtDUF707, AtKAN1, and AtEML2 by VPg-Pro *in vitro* to release the expected cleaved fragments (Fig. 3D). The AtPRC protein, which was not cleaved by PPV Pro, was also not cleaved by VPg-Pro (Fig. 3D).

### Differential *in vitro* cleavage of selected plant proteins by the PPV, TuMV, and TEV NIa proteases.

We also tested the cleavage of candidate proteins by the TuMV and TEV proteases (Fig. 3A). All proteins that were shown to be cleaved by PPV Pro included a Q at the P1 position, H at the P2 position, and V at the P4 position and were also cleaved by TuMV Pro. Because most of these proteins did not include an E at the P6 position and/or a Y at the P3 position, which are strongly preferred by TEV Pro, they were not cleaved by this protease. Interestingly, PpP100 was cleaved by all three proteases (Fig. 3A, lanes 34 to 36), as the cleavage site included the conserved residues V at the P4 position and H at the P2 position preferred by the PPV and TuMV proteases, as well as the conserved residues E at the P6 position and Y at the P3 position favored by the TEV protease. The AtPRC protein, which was not cleaved by PPV Pro or VPg-Pro, was not cleaved efficiently by the TuMV or TEV proteases either, although a faint band of approximately 30 kDa detected after incubation with the TuMV Pro may correspond to the expected 27.5-kDa cleaved fragment (Fig. 3A, lane 19). AtFbKr and PpRHLP, which were only inefficiently cleaved by PPV Pro, were also only partially cleaved by TuMV Pro (Fig. 3A, lanes 15 and 43).

We noted differences in the relative cleavage efficiency of the different substrates by the PPV and TuMV proteases. For example, PPV Pro cleaved AtGPI-AALP more effectively than TuMV Pro did (Fig. 3A, lanes 30 and 31). Conversely, TuMV Pro cleaved AtDUF707, PpDDB, and PpCESA2 more effectively than PPV Pro did (Fig. 3A, lanes 26 and 27, 38 and 39, and 22 and 23). The reasons for these differences are not clear. The presence of G at the P1′ position and N at the P6 position of the AtGPI-AALP cleavage site is consistent with the PPV cleavage site consensus sequence but not with the TuMV cleavage site consensus sequence, possibly explaining the more efficient cleavage of this protein by PPV Pro. On the other hand, these amino acids are also present in the PpDDB cleavage site, which is cleaved more efficiently by TuMV Pro. Other factors, including the nature of amino acids at the P3 and P5 positions or at other positions beyond the P6 to P1′ positions and/or the presentation of the cleavage site in the tertiary structure of the protein could impact the recognition of these cleavage sites by various proteases.

### *In vivo* cleavage of AtEML2 by the PPV and TuMV NIa proteases.

**FIG 4:**
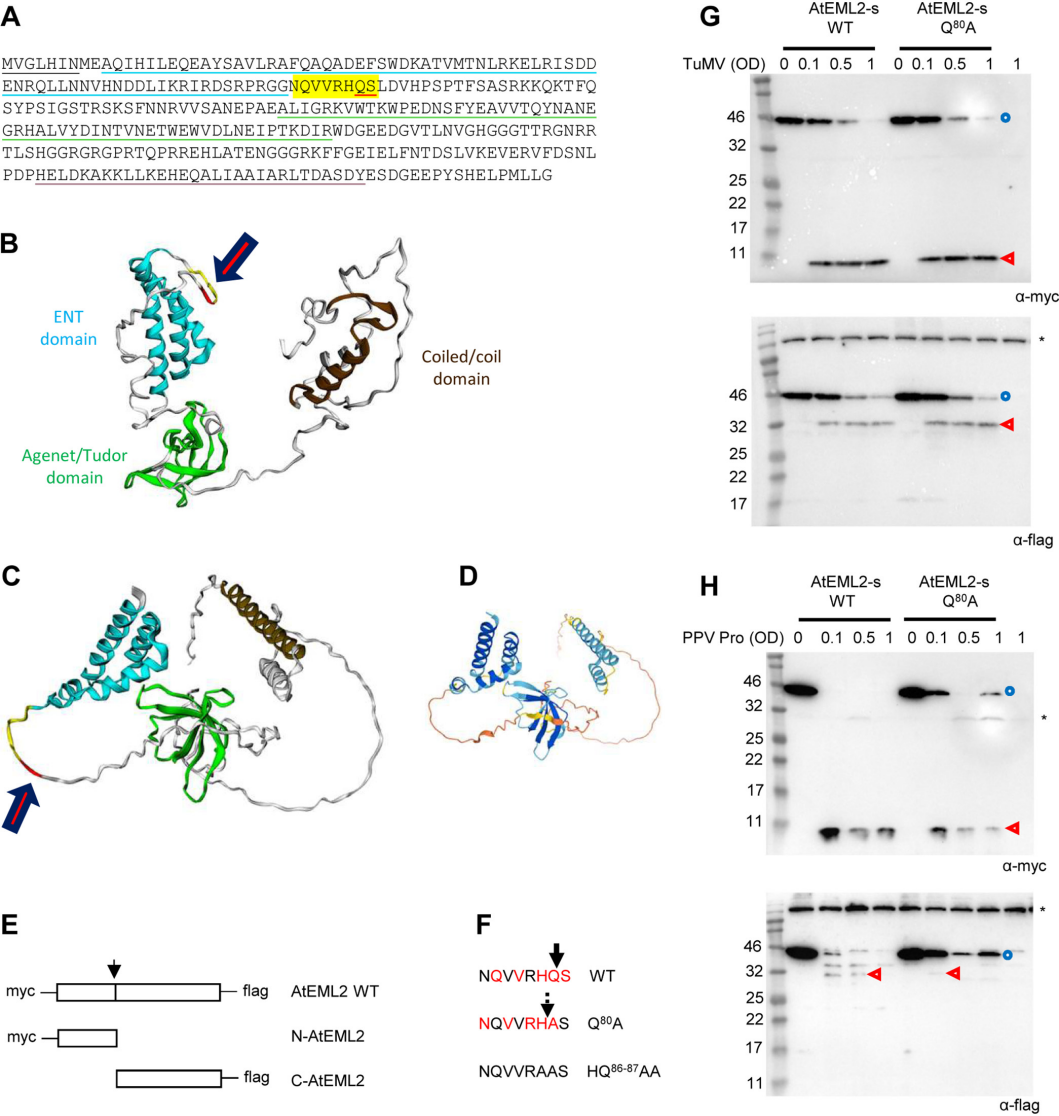
*In vivo* cleavage of the smaller isoform of the AtEML2 protein (AtEML2-s). (A) Sequence of AtEML2-l (large isoform produced from a predominant splice variant). Please note that the sequence of the smaller isoform, AtEML2-s, is identical to that of the AtEML2-l isoform but lacks the first 7 amino acids (underlined in black). Amino acids corresponding to the ENT domain, Agenet/Tudor domain, and a predicted coiled-coil domain are underlined in blue, green, and brown, respectively. Letters highlighted in yellow correspond to the P6 to P1′ positions of the predicted cleavage site, with the P1 and P1′ positions underlined in red. (B to D) Structural models of the AtEML2-s protein predicted using Phyre2 (see Materials and Methods) (B) or AlphaFold (PDB file of the model available at https://www.alphafold.ebi.ac.uk/) (C). For both models, the color scheme is as follows. ENT domain in blue, Agenet/Tudor domain in green, coiled-coiled domain in brown, P6 to P2 positions of the predicted cleavage site in yellow, and P1 to P1′ position of the cleavage site in red. The position of the cleavage site is indicated with the red arrow. The degree of confidence in the Phyre2 model (B) differed for the domains of the protein. The ENT domain was modeled with a very high degree of confidence (99.8%), based in part on the solved structure of the ENT domain from human EMSY (PDB ID 2FMM). The Agenet/Tudor domain was modeled with a high degree of confidence (87.9%), based in part on the solved structure of the Tudor domain of the tumor suppressor protein p53-binding protein 1 (PDB ID 1XNI). The coiled-coiled domain was modeled with a medium degree of confidence (74%), based in part on the solved structure of a similar domain in the tubulin folding cofactor a (chaperone a) of *A. thaliana* (PDB ID 3MXZ). Other regions of the protein were predicted to be disordered, and their exact positioning within the three-dimensional (3D) structure of the protein could only be modeled *ab silico* with a low degree of confidence. The degree of confidence in the AlphaFold model (C) also differed by region of the protein (D), shown by the following color scheme: dark blue (more than 90%), light blue (70 to 90%), yellow (50 to 70%), and orange (less than 50%). In both models, the predicted cleavage site is contained in a flexible linker located between the ENT and Agenet/Tudor domains. (E) Schematic representation of AtEML2 protein fused to the Myc and Flag epitopes. Experiments were conducted using the two isoforms AtEML2-s (G, H) and AtEML2-l (see Fig. 5). The position of the predicted cleavage site is shown by the black arrow. Cleavage by the NIa protease would release the Myc-tagged N-terminal fragment (N-AtEML2) and the Flag-tagged C-terminal fragment (C-AtEML2). (F) Sequence of wild-type or mutated AtEML2 cleavage site. The position of the predicted cleavage is indicated by the thick black arrow above the wild-type sequence, with conserved amino acids at the P6, P4, P2, P1, and P1′ positions shown in red. A possible alternate cleavage event is shown with the broken arrow in the Q^80^A mutant, with the shifted P6, P4, P2, P1, and P1′ positions shown in red. Cleavage was not observed for the HQ^86-87^AA mutant (see Fig. 5). (G) Cleavage of AtEML2-s by the NIa protease released from the TuMV polyprotein. N. benthamiana leaves were agroinfiltrated with a mixture of A. tumefaciens transformed with the AtEML2-s constructs (WT or Q^80^A mutant; optical density [OD] = 1) and A. tumefaciens transformed with the TuMV infectious clone (OD = 0.1 to 1, as indicated above each lane). Control experiments did not incorporate A. tumefaciens transformed with the TuMV infectious clone (marked as OD = 0). (H) Cleavage of AtEML2-s by PPV Pro expressed ectopically. N. benthamiana leaves were agroinfiltrated with a mixture of A. tumefaciens transformed with the AtEML2-s constructs (WT or Q^80^A mutant; OD = 1) and A. tumefaciens transformed with the PPV Pro construct (OD = 0.1 to 1, as indicated above each lane). Control experiments did not incorporate A. tumefaciens transformed with the PPV Pro construct (marked as OD = 0). (G and H) Plant protein extracts were separated by 12% SDS-PAGE and transferred to PVDF membranes. Immunoblots shown at the top were probed with anti-Myc antibodies, and those at the bottom with anti-Flag antibodies. The blue circles and red arrowheads indicate the full-length protein and cleavage products, respectively. Asterisks (*) indicate background bands that are present in all lanes, including control lanes that corresponded to extracts from leaves that were not expressing AtEML2-s.

Next, we wished to confirm that identified plant proteins were also cleaved by potyvirus NIa proteases *in vivo*. We chose to test AtEML2, which was efficiently cleaved *in vitro* by both the PPV and TuMV proteases. AtEML2, a nuclear protein, belongs to a family of histone readers (also including AtEML1, AtEML3, and AtEML4) that have been associated with postfertilization seed development and pathogen resistance, including resistance to geminiviruses (69–71). The cleavage site is not conserved in AtEML1, AtEML3, or AtEML4. The putative AtEML2 cleavage site is located between the ENT and Agenet/Tudor-like domains (Fig. 4A). Models of the protein structure (predicted using the Phyre2 or AlphaFold methods) map the cleavage site to a predicted flexible arm (disordered region) joining the two domains (Fig. 4B to D), suggesting that it should be readily accessible to the nuclearly localized Pro and/or VPg-Pro. We first attempted to produce antibodies against peptides derived from either the N-terminal or C-terminal portions of the protein. Unfortunately, we were unable to detect the endogenous AtEML2 protein in extracts from healthy or TuMV-infected *A. thaliana* plants with these antibodies, possibly because the protein only accumulates in low concentrations.

We designed an alternate approach in which we transiently expressed a version of AtEML2 that is fused to the Myc epitope tag at its N terminus and to the Flag epitope tag at its C terminus (Fig. 4E). There are two isoforms of AtEML2, produced by alternative splicing of the mRNA. The two isoforms differ by 7 amino acids at their N terminus (Fig. 4A). In initial experiments, we used the smaller isoform (AtEML2-s, AT5G06780.2, NCBI accession number NP_001190242.1), which was also the isoform that was confirmed to be cleaved *in vitro*. Using agroinfiltration, we expressed AtEML2-s in N. benthamiana leaves. The anticipated full-length protein (calculated molecular mass of 39.7 kDa, including fusion to Myc and Flag epitope tags and vector sequences) was readily detected in leaf extracts by both anti-Myc and anti-Flag antibodies, although it migrated slightly slower than anticipated, just below the 46-kDa marker (Fig. 4G). To test for cleavage by potyvirus proteases, we used two approaches. In the first approach, we launched TuMV infection at the same time as we launched AtEML2-s expression by coagroinfiltrating Agrobacterium tumefaciens transformed with an infectious TuMV clone whose expression is driven by the 35S promoter (72). In this approach, the NIa protease should be released from the viral polyprotein by autocatalytic cleavage, allowing the accumulation of 6K2-VPg-Pro, VPg-Pro, and Pro in infiltrated leaves. In the presence of the TuMV infectious clone, disappearance of the full-length AtEML2 protein was observed, along with the appearance of the expected N-terminal fragment (calculated molecular mass of 12.3 kDa, detected by the anti-Myc antibody and migrating slightly faster than anticipated, just below the 11-Da marker) and C-terminal fragment (calculated molecular mass of 27.2 kDa, detected by the anti-Flag antibody and migrating slightly slower than anticipated, just below the 32-kDa marker) (Fig. 4G). In the second approach, we coexpressed AtEML2-s with PPV Pro. We mixed A. tumefaciens transformed with the PPV Pro construct with A. tumefaciens transformed with the AtEML2-s construct. In this approach, only the mature Pro will accumulate in plants. Cleavage of AtEML2-s was also clearly detected with this approach (Fig. 4H). The Myc-tagged N-terminal fragment was readily detected in both experimental set-ups. On the other hand, the Flag-tagged C-terminal fragment was more difficult to detect and did not accumulate to very high levels, suggesting that it was less stable (Fig. 4G and H).

We produced a mutant derivative of AtEML2-s in which the Q at the P1 position of the predicted cleavage site was mutated to A (mutant Q^80^A, numbering from the first amino acid of the AtEML2-s protein) (Fig. 4F). This mutation was predicted to eliminate the cleavage. Surprisingly however, cleavage of this mutant was still detected in both experimental set-ups (Fig. 4G and H), although cleavage of the AtEML2-s Q^80^A mutant by PPV Pro was less efficient than that of AtEML2-s WT (Fig. 4H). There was no significant shift in the size of the cleaved fragments with the Q^80^A mutant, suggesting that cleavage of the WT and Q^80^A mutants occurred at or near the same position. Cleavage at an A/S site would be highly unusual and has not been reported for NIa proteases in the family *Potyviridae* (30). On the other hand, our analysis of cleavage sites in TuMV and PPV polyproteins suggested some flexibility at the P1 position, with some cleavage sites presenting P, D, or R at this position (Table 1). We hypothesize that the cleavage may have shifted by one amino acid in the Q^80^A mutant, resulting in the alternate NQVVRH/A cleavage site (Fig. 4F). This proposed alternate cleavage site would still include a V at the P4 position, which is strongly preferred in PPV and TuMV cleavage sites (Table 1). It also contains N at the P6 position. Our *in vitro* experiments showed that cleavage sites with N at the P6 position are efficiently cleaved by both the TuMV and PPV proteases (Fig. 3A). The H at the P1 position of the proposed alternate cleavage site is unusual, but there are some precedents in the family *Potyviridae*. Indeed, H/A cleavage sites have been proposed for sweet potato mild mottle virus (a member of the genus Ipomovirus) (30). Finally, the R at the P2 position, although not found in PPV or TuMV polyprotein cleavage sites, shows a charge conservation with the H normally found at that position. Our analysis of PPV and TuMV cleavage sites also suggested some flexibility in the nature of the amino acid present in the P2 position (Table 1).

**FIG 5:**
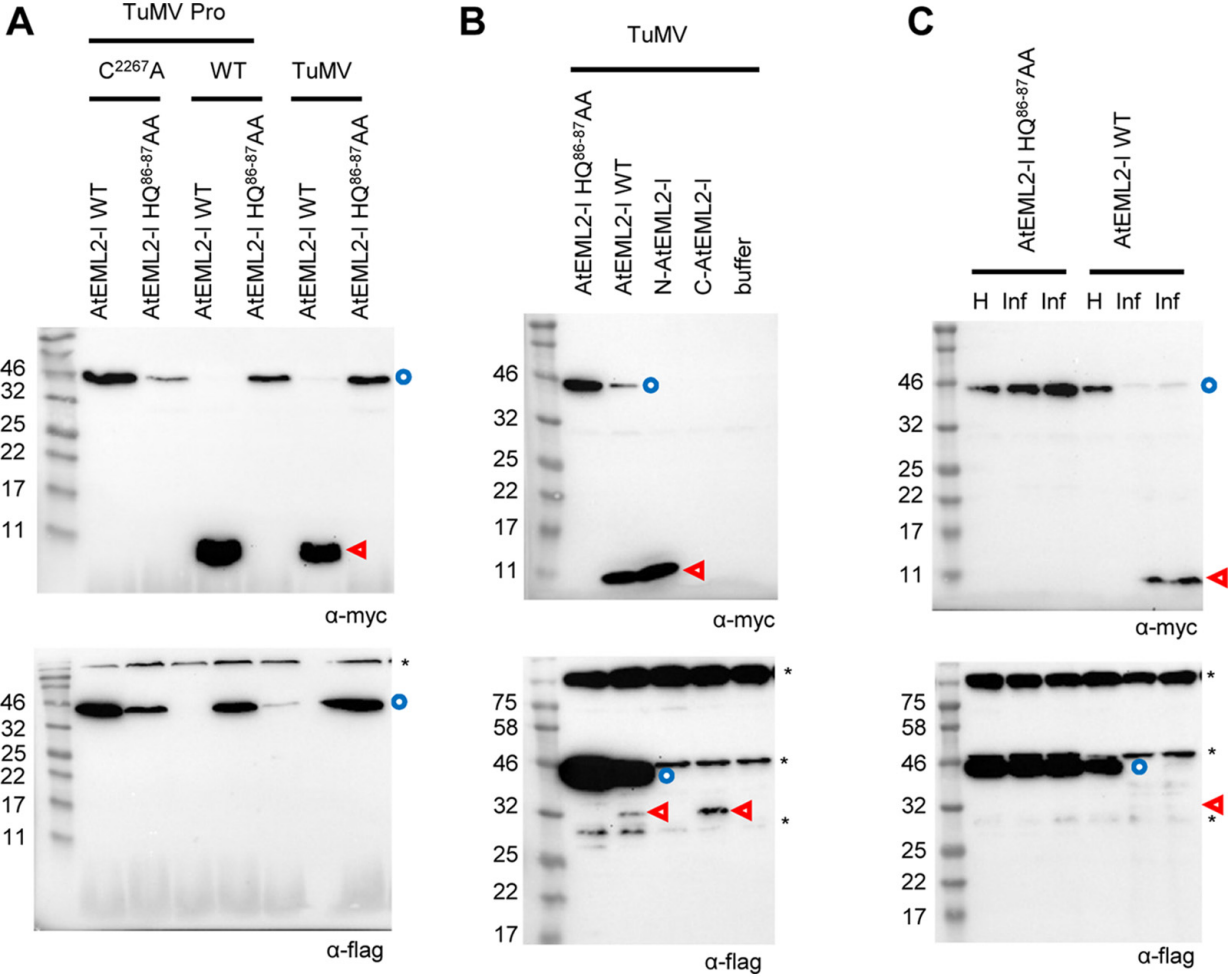
*In vivo* cleavage of the larger isoform of AtEML2 (AtEML2-l). (A) Cleavage of AtEML2-l by TuMV Pro expressed ectopically or released from the TuMV polyprotein. N. benthamiana leaves were agroinfiltrated with mixtures of A. tumefaciens as indicated above each lane. These included A. tumefaciens transformed with the AtEML2-l constructs (WT or HQ^86-87^AA mutant; OD = 1) mixed with either A. tumefaciens transformed with the TuMV Pro construct (WT or C^2267^A mutant; OD = 0.2) or A. tumefaciens transformed with the TuMV infectious clone (OD = 1). (B) Cleavage of AtEML2-l by Pro released from the TuMV polyprotein. N. benthamiana leaves were agroinfiltrated with a mixture of A. tumefaciens transformed with the AtEML2-l constructs (WT, HQ^86-87^AA mutant, or the individual N-terminal or C-terminal fragments; OD = 0.5) and A. tumefaciens transformed with the TuMV infectious clones (OD = 0.25). (C) Cleavage of AtEML2-l in TuMV-infected plants. Plants were first inoculated with extracts from TuMV-infected plants. Ten days later, upper leaves from healthy control plants (H) or TuMV-infected plants (Inf) were agroinfiltrated with A. tumefaciens transformed with the AtEML2-l constructs (WT or HQ^86-87^AA mutant; OD = 0.5). (A to C) Plant protein extracts were separated by 12% SDS-PAGE and transferred to PVDF membranes. Immunoblots shown at the top were probed with anti-Myc antibodies and those at the bottom with anti-Flag antibodies. Blue circles and red arrowheads indicate the full-length protein and cleavage products, respectively. Asterisks (*) indicate background bands that are present in all lanes, including control lanes that corresponded to extracts from leaves that were not expressing AtEML2-l.

To confirm cleavage of all isoforms of AtEML2 *in vivo*, we produced new clones that used the sequence of the larger isoform (AtEML2-l, AT5G06780.1). Coexpression of Myc- and Flag-tagged AtEML2-l together with the wild-type TuMV Pro resulted in cleavage of the protein and accumulation of the Myc-tagged N-terminal fragment (Fig. 5A). The Flag-tagged C-terminal fragment was not clearly detected in this experiment. Coexpression of AtEML2-l with a catalytically inactive version of the protease (TuMV Pro C^2267^A) did not result in any cleavage, confirming the specific processing of AtEML2-l by the wild-type TuMV Pro (Fig. 5A). As observed for AtEML2-s, cleavage of AtEML2-l was also observed in plants coinfiltrated with A. tumefaciens containing the TuMV infectious clone (Fig. 5A).

We introduced a new cleavage site mutation in clone AtEML2-l, this time mutating both the Q at the P1 position and the H at the P2 position to A (mutant HQ^86-87^AA, numbering from the first amino acid of the AtEML2-l protein; see Fig. 4F). The AtEML2-l HQ^86-87^AA mutant was not cleaved by TuMV Pro (Fig. 5A). To further confirm that cleavage was indeed occurring at the predicted cleavage site, we produced new clones that allowed the expression of the expected Myc-tagged N-terminal fragment (N-AtEML2-l) or of the Flag-tagged C-terminal fragment (C-AtEML2-l) (Fig. 4E). As can be seen in Fig. 5B, the N-terminal fragment released from the wild-type AtEML2-l comigrated with the expressed N-AtEML2-l protein. Similarly, the C-terminal fragment released from the wild-type AtEML2-l comigrated with the expressed C-AtEML2-l protein. Taken together, these results confirm that both isoforms of AtEML2 can be cleaved by TuMV Pro and PPV Pro at the predicted NQVVRHQ/S cleavage site *in vivo*.

In the experimental designs described above, expression of the mature PPV or TuMV Pro or that of the entire TuMV polyprotein is driven by the 35S promoter. Next, we wished to confirm that AtEML2 could be cleaved in the context of a natural virus infection. We used extracts from TuMV-infected plants to inoculate new N. benthamiana plants. At 10 days postinfection, upper symptomatic leaves were agroinfiltrated to allow the expression of AtEML2-l WT or HQ^86-87^AA (Fig. 5C). The wild-type AtEML2-l protein was cleaved in TuMV-infected leaves, but not in healthy leaves. In contrast, the HQ^86-87^AA mutant was not cleaved in virus-infected leaves. This experiment confirmed that one or several forms of the NIa protease expressed during a natural TuMV infection are able to cleave the transiently expressed AtEML2-l protein.

### Partial *in vivo* cleavage of AtDUF707 by the PPV and TuMV NIa proteases.

**FIG 6:**
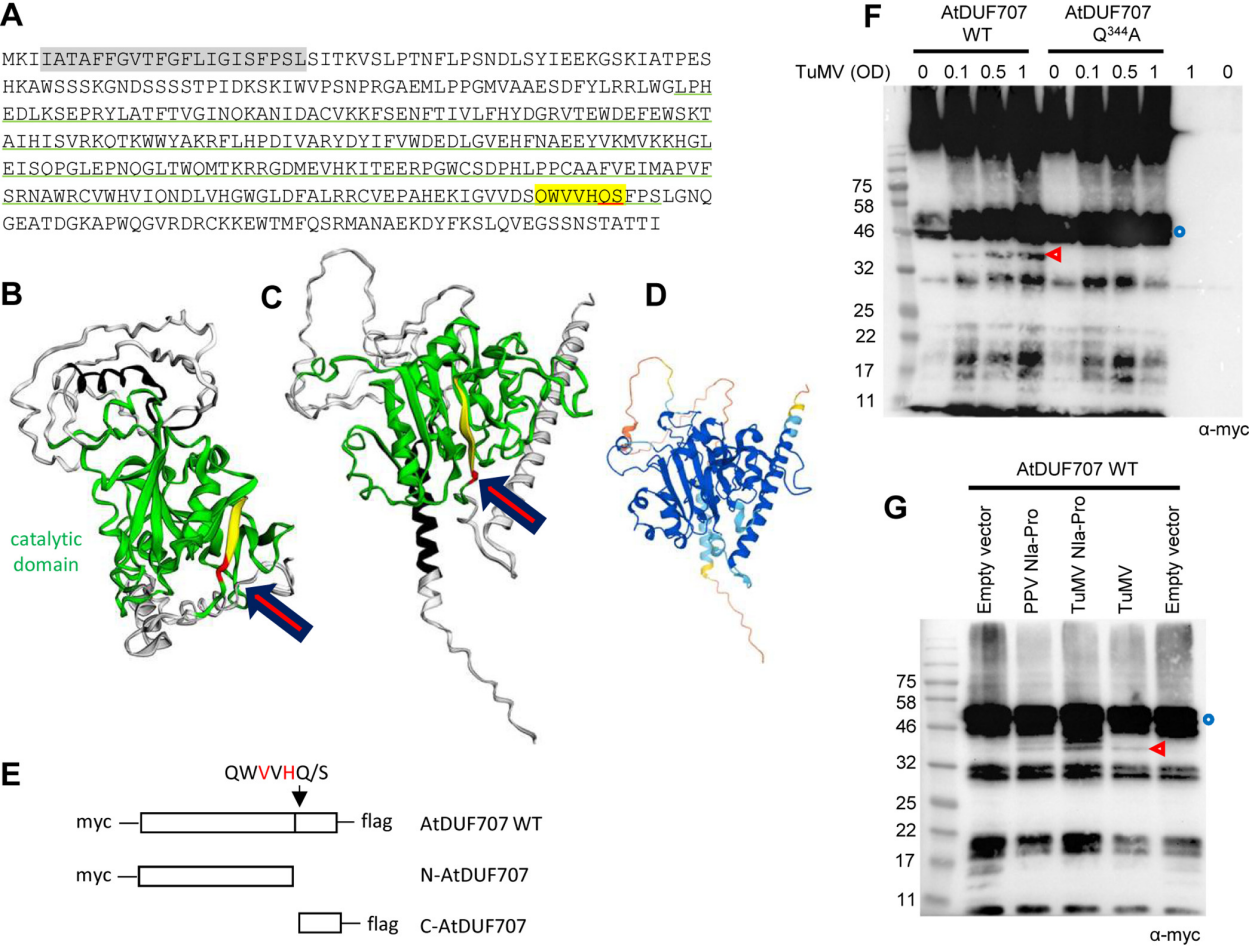
Partial *in vivo* cleavage of AtDUF707. (A) Sequence of AtDUF707. The predicted catalytic domain is underlined in green. A strongly predicted *trans*-membrane helix is highlighted in gray. Letters highlighted in yellow correspond to the P6 to P1′ positions of the predicted cleavage site with the P1 and P1′ positions underlined in red. (B to D) Structural models of the AtDUF707 protein predicted using Phyre2 (see Materials and Methods) (B) or AlphaFold (PDB file of the model available at https://www.alphafold.ebi.ac.uk/) (C). For both models, the color scheme is as follows: catalytic domain in green, putative *trans*-membrane helix in black, P6 to P2 positions of the predicted cleavage site in yellow, and P1 to P1′ position of the cleavage site in red. The position of the cleavage site is indicated by the red arrow. The degree of confidence in the Phyre2 model (B) differed by domain of the protein. The predicted catalytic domain was modeled with a high degree of confidence (90.4%), based in part on the solved structure of the catalytic domain from ATP synthase subunits of orf6 of the f1-atpase2 operon of Rhodobacter blasticus (PDB ID 2QGI) and on those of several other transferase domains from acetylgalactosaminyltransferases. Other regions of the protein were not modeled with a high degree of confidence. The degree of confidence in the AlphaFold model (C) also differed by region of the polyprotein (color scheme as in Fig. 4D) (D). In both models, the cleavage site is located at the base of a β-sheet, which is part of the predicted catalytic domain. (E) Schematic representation of AtDUF707 protein fused to the Myc and Flag epitopes. The predicted cleavage site is shown by the black arrow. Cleavage by the NIa protease would release the Myc-tagged N-terminal fragment (N-AtDUF707) and the Flag-tagged C-terminal fragment (C-AtDUF707). (F) Cleavage of AtDUF707 by the NIa protease released from the TuMV polyprotein. N. benthamiana leaves were agroinfiltrated with a mixture of A. tumefaciens transformed with the AtDUF707 constructs (WT or Q^344^A mutant) (OD = 1.0) and A. tumefaciens transformed with the TuMV infectious clone (OD = 0.1 to 1, as indicated above each lane). Control experiments did not incorporate A. tumefaciens transformed with the TuMV infectious clone (marked as OD = 0). (G) Cleavage of DUF707 by PPV or TuMV Pro[s]. N. benthamiana leaves were agroinfiltrated with a mixture of A. tumefaciens transformed with the AtDUF707 WT construct (OD = 0.5) and A. tumefaciens transformed with the empty vector (OD = 0.1), PPV Pro construct (OD = 0.1), TuMV Pro construct (OD = 0.1), or TuMV infectious clone (OD = 1). (F and G) Plant protein extracts were separated by 12% SDS-PAGE and transferred to PVDF membranes. Immunoblots were probed with anti-Myc antibodies. Blue circles and red arrowheads indicate the full-length protein and cleavage products, respectively. The large bands at the top of the gel in panel F are likely due to aggregation of the protein.

Next, we tested whether AtDUF707, a membrane protein, can also be cleaved by the PPV and/or TuMV NIa proteases *in vivo*. Annotated as lysine ketoglutarate reductase *trans*-splicing protein, AtDUF707 is poorly characterized. A putative glycosyl transferase catalytic domain was identified, and the cleavage site is located immediately downstream of this domain (Fig. 6A to D). The subcellular localization of this protein is annotated as chloroplastic in TAIR based on *in silico* prediction using AtSubP. Plant-mPLoc also predicted a chloroplast localization, while LocTree3 predicted an association with the inner mitochondrial membrane, and TargetP predicted the presence of an N-terminal endoplasmic reticulum signal peptide. While the exact subcellular localization of this protein is not known, a *trans*-membrane domain is strongly predicted in its N terminus.

We used the same strategy described above for AtEML2 and transiently expressed Myc- and Flag-tagged AtDUF707 by agroinfiltration of N. benthamiana leaves (Fig. 6E). A protein with a migration consistent with the calculated molecular mass of the fusion protein (48.2 kDa) was readily detected (Fig. 6F). Coagroinfiltration with increasing amounts of A. tumefaciens transformed with the TuMV infectious clone led to the release of a band of approximately 40 kDa that was detected by the anti-Myc antibodies and was consistent with the calculated molecular mass of the N-terminal cleaved fragment (40.6 kDa). We also tested a mutant of AtDUF707 with a mutation of the Q at the P1 position of the predicted cleavage site to A (Q^344^A). We showed above that this mutant is not cleaved by PPV Pro *in vitro* (Fig. 3B), and consistently, this mutant was not cleaved in the presence of TuMV infectious clones *in vivo* either (Fig. 6F). These results suggested that one or several forms of the TuMV NIa protease released from the TuMV polyprotein could cleave the tagged AtDUF707 protein at the predicted cleavage site. However, the *in vivo* cleavage in the presence of TuMV infectious clones was much less efficient than that observed for AtEML2 (compare Fig. 6F to Fig. 4G; please note that both experiments were conducted in parallel with the same batches of plants and A. tumefaciens transformed with the TuMV infectious clone). Next, we tested whether the mature PPV Pro or TuMV Pro expressed ectopically could also cleave AtDUF707. Both PPV Pro and TuMV Pro cleaved AtDUF707 in agroinfiltrated leaves, but, as above, the cleavage was inefficient (Fig. 6G), and only small amounts of the N-terminal cleaved fragment were detected. The small C-terminal fragment was never detected by the anti-Flag antibodies in spite of repeated attempts, and it is possible that it is rapidly degraded. It is interesting to note that although cleavage of AtDUF707 by TuMV Pro was efficient *in vitro* (Fig. 3A), it was only partial *in vivo* (Fig. 6F and G). It is likely that the cleavage site is not easily accessible to the NIa protease in plant cells, possibly because of the subcellular localization and/or membrane topology of the protein. Given the inefficient *in vivo* cleavage of this protein, we did not pursue further experiments.

## DISCUSSION

That host proteins are cleaved by viral proteases to facilitate virus infection is well established for vertebrate viruses (9–14). Here, we show that potyvirus proteases can also target plant proteins. We used a bioinformatics approach to identify putative cleavage sites in the proteomes of three natural and experimental hosts of PPV. Using a combination of *in vitro* and *in vivo* approaches, we confirmed the cleavage of several plant proteins by the PPV and TuMV NIa proteases.

The bioinformatics approach used to identify cleavage events was facilitated by the stringent cleavage site specificity of potyviral NIa proteases (30). Indeed, the cleavage site consensus sequence [EQN]xVxH[QE]/[STGA] for the PPV NIa protease includes conserved amino acids at the P1′, P1, P2, P4, and P6 positions. However, we acknowledge that some PPV polyprotein cleavage sites deviate from this consensus (Table 1). We also obtained experimental evidence for unexpected flexibility in the PPV and TuMV proteases specificities. Indeed, we observed that a cleavage site mutant of AtEML2, lacking a Q or E at the P1 position, was cleaved by the PPV and TuMV proteases, although at a reduced efficiency (Fig. 4). We suggested that cleavage of this mutant occurred at a cryptic cleavage site, created by the mutation, that includes a H at the P1 position and an R at the P2 position but maintains the canonical V at the P4 position, A at the P1′ position, and N at the P6 position. We conclude that although the use of strict search terms that match the PPV cleavage site consensus sequence identified putative cleavage events in the plant proteome, it will not allow the discovery of suboptimal cleavage events at sites that deviate from the strict consensus sequence but may still have an important biological impact. It may be possible in the future to identify additional cleavage events by adjusting (loosening) the search parameters.

In recent studies, proteomic approaches have been used to selectively label and identify host protein fragments released after proteolytic cleavage by viral proteases, including the subtiligase labeling approach (18) or N-terminal amine isotopic labeling (N-TAILS) (17). These approaches are powerful in that they are unbiased and allow the identification of all cleavage events, including those that do not fit the exact cleavage site consensus sequence of viral proteases. On the other hand, since proteomic approaches depend on the selective enrichment of cleaved fragments, they also identify protein fragments generated by host protease cleavage or by unspecific degradation. In addition, cleaved fragments must be in sufficient amounts to allow their detection by proteomic methods. In contrast, the bioinformatics approach described here depends on the scanning of the entire plant proteome for specific search terms and is not influenced by the relative abundance of the target protein and of the cleaved protein fragments, many of which could be unstable, as shown for the AtEML2 C-terminal fragment (Fig. 4 and 5). In future experiments, combining proteomic and *in silico* approaches should help obtain a more exhaustive list of host proteins targeted by potyvirus proteases.

**FIG 7:**
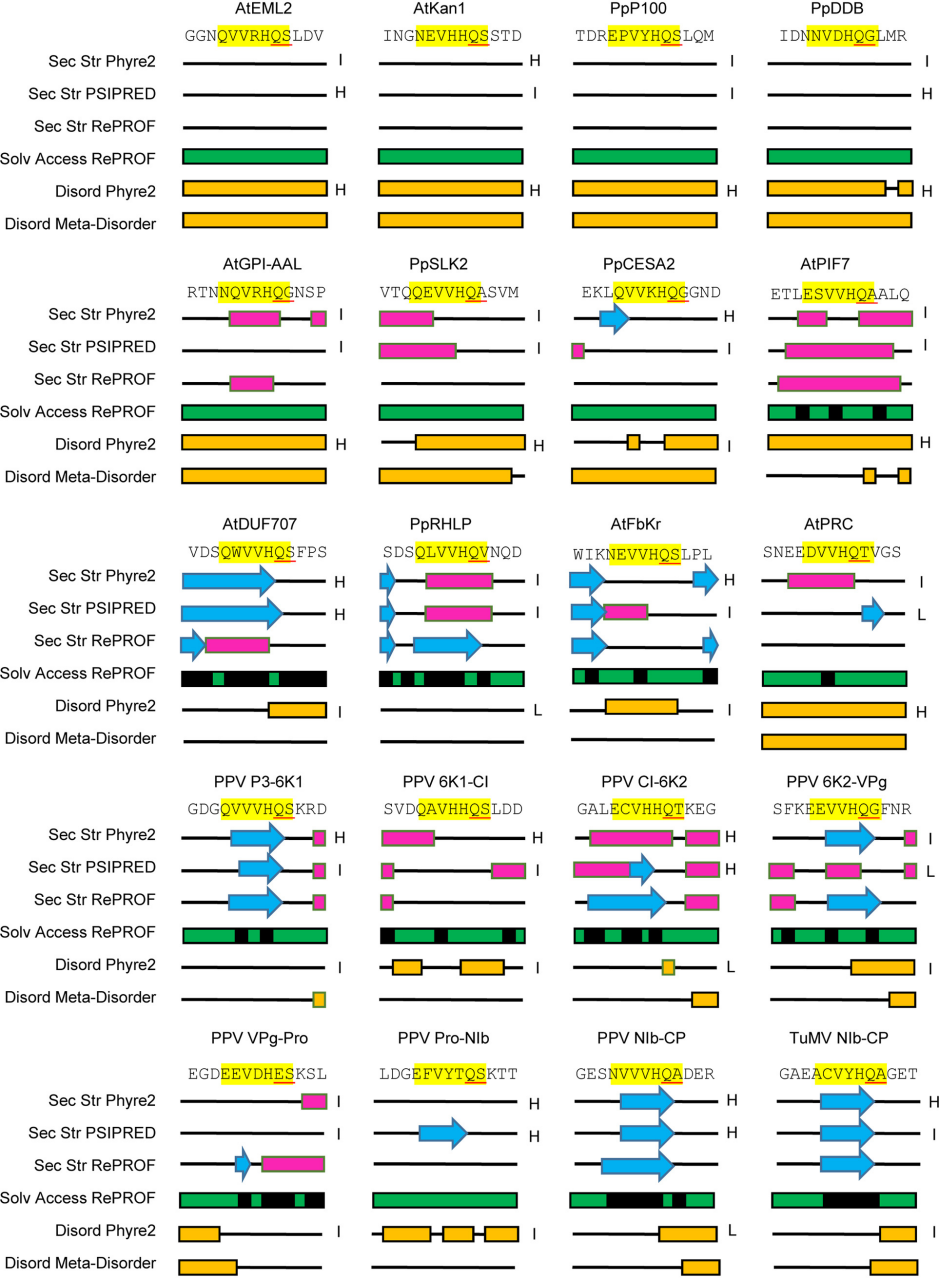
Predicted secondary structure and solvent accessibility for the region around the predicted cleavage site of selected plant proteins and around known cleavage sites of the PPV and TuMV polyproteins. The amino acid sequence is shown at the top with the P6 to P1′ positions of the cleavage site highlighted in yellow and the P1 and P1′ positions underlined in red. Secondary structure (Sec Str) was predicted using Phyre2, PSIPRED, and RePROF (see Materials and Methods). Regions with predicted α-helices and β-strands are shown with pink boxes and blue arrows, respectively. Solvent accessibility (Solv Access) was predicted using RePROF. Green and black indicate exposed and hidden regions of the protein, respectively. Regions of the proteins with disorder state (Disord, shown in yellow) were predicted using Phyre2 and Meta-Disorder. The degree of confidence in the RePROF and Meta-Disorder models is reported to be 70% on average (individual confidence levels are not provided for each prediction). For PSIPRED Sec Str predictions and Phyre2 Sec Str and Disord predictions, levels of confidence are provided for each individual amino acids. The overall level of confidence in the models was estimated by averaging the levels of confidence for the 13 amino acids considered for each cleavage site (positions P9 to P4′). For PSIPRED, the scale was from 1 to 8. Average scores from 1 to 3 were considered a low level of confidence. Average scores from 4 to 5 were labeled as an intermediate level of confidence, and average scores from 6 to 8 were marked as a high level of confidence. For Phyre2, the scale was from 0 to 9. Average scores of 0 to 2, 3 to 6, and 7 to 9 were labeled as low, intermediate, and high levels of confidence, respectively. The estimated overall levels of confidence are indicated as high (H), intermediate (I), or low (L) on the right of each Phyre2 or PSIPRED prediction.

The strict consensus sequence used to interrogate the plant proteome allowed the identification of approximately 100 putative target proteins for each proteome, several of which were experimentally confirmed to be cleaved by the PPV (and TuMV) proteases. Indeed, of the 12 plant proteins tested, all but one (AtPRC) were cleaved by PPV Pro *in vitro*. Most were efficiently cleaved under our experimental conditions, although two were only partially cleaved (PpRHLP and AtFbKr). All plant proteins tested (with the exception of PpRHLP, which had a variation at the P1′ position) were an exact fit to the determined cleavage site specificity of the PPV Pro. In fact, the inefficiently cleaved AtFbKr cleavage site sequence (NEVVHQ/S, P6 to P1′ position) was identical to that of AtKAN1, which was efficiently cleaved. As demonstrated more than 30 years ago for poliovirus polyprotein cleavage sites, efficient proteolytic processing requires not only a good fit of the cleavage site to the consensus sequence but also optimal presentation of the cleavage site in the three-dimensional structure of the target protein (73). Thus, presence of a cleavage site in flexible linkers or disordered regions of the protein should facilitate efficient processing. We used a computational approach to predict secondary structure, disorder and solvent accessibility of each tested cleavage site using the P9 to P4′ sequence of these sites (Fig. 7). Many of the efficiently processed cleavage sites were predicted to be in unstructured and/or disordered regions of the proteins (AtEML2, AtKAN1, PpP100, PpDDB, AtGPI-AAL, pSLK2, and PpCESA2). In contrast, two of the poorly cleaved sites (PpRHLP and AtFbKr) were predicted to be in more structured regions of the protein that may not be readily accessible. However, there were exceptions to this general trend. Indeed, two cleavage sites that were readily processed *in vitro* (AtPIF7 and AtDUF707) were not confidently predicted to be in unstructured or disordered regions of the protein by all software tools. In addition, AtPRC, which was not cleaved by the PPV protease *in vitro* was not predicted to be in a strongly structured region of the protein. Finally, the secondary structure predictions alone were not able to confidently demonstrate accessibility of the known PPV polyprotein cleavage sites to the protease (Fig. 7).

**FIG 8:**
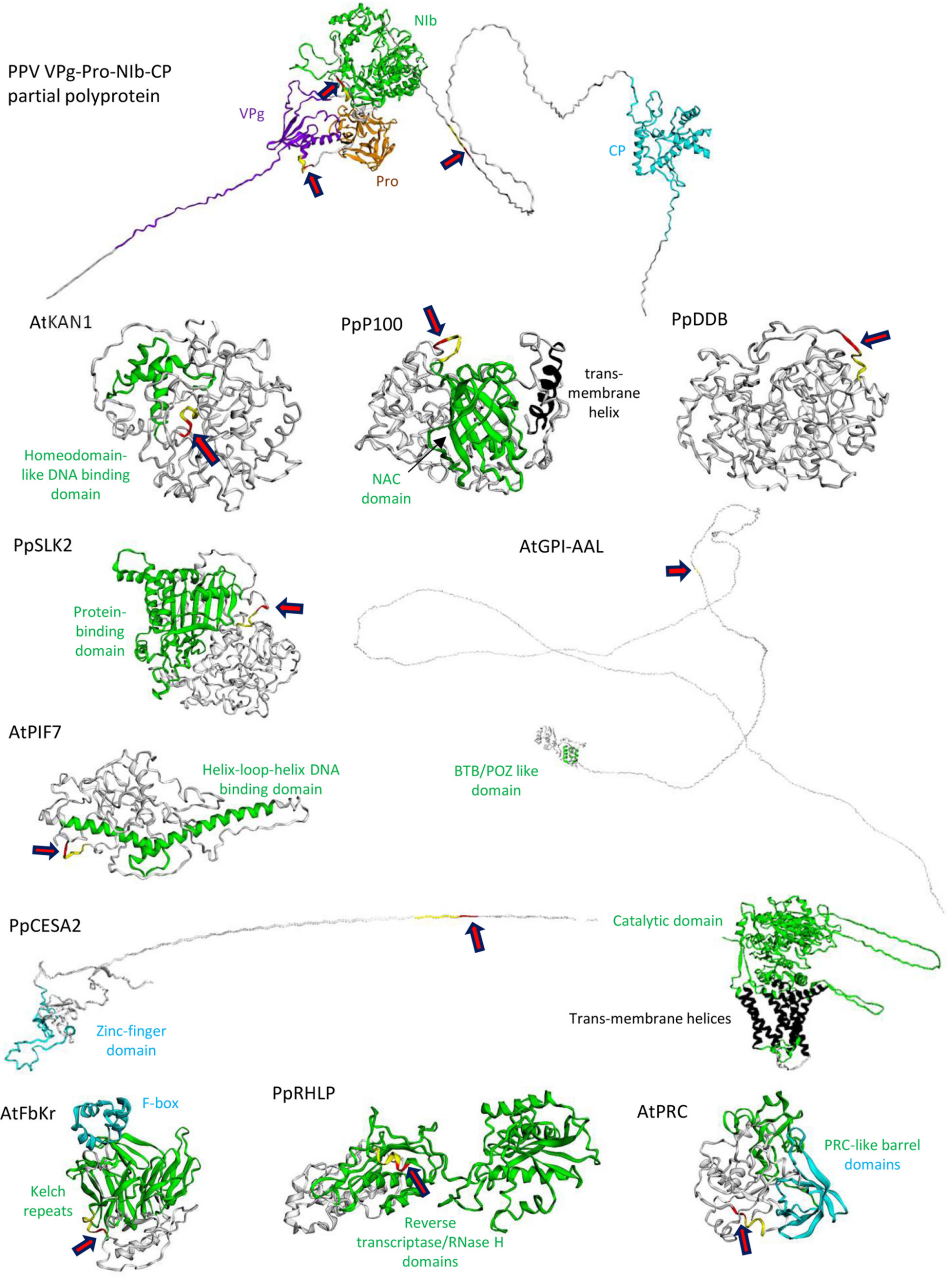
Structural models for the partial viral polyprotein and for selected plant proteins. For each protein, structural models were produced using Phyre2. The degree of confidence in the models differed by domain of each protein. Domains predicted with a stronger degree of confidence based on the resolved structure of similar structural domains are highlighted in color. Regions shown in white were not modeled with a high degree of confidence. Please see Fig. S1 to S11 in the supplemental material for more details. For each protein, the position of the predicted cleavage site (P6 to P2 positions highlighted in yellow; P1 and P1′ positions highlighted in red) is shown with a red arrow.

Homology modeling of the three-dimensional structure of a partial PPV polyprotein (VPg-Pro-NIb-CP; model based on the solved structures of related VPg, Pro, NIb, and CP domains) using Phyre2 was more informative, in that it highlighted the position of the cleavage sites in flexible linkers joining the different protein domains (Fig. 8; see also Fig. S1 in the supplemental material). Although not all regions of the target plant proteins could be modeled with a high degree of confidence, Phyre2 modeling predicted that most cleavage sites were located in unstructured regions of the target proteins (AtEML2, Fig. 4B; AtKAN1, PpP100, PpDDB, PpSLK2, AtGPI-AAL, AtPIF7, and PpCESA2, Fig. 8 and Fig. S2 to S8). Comparison of publicly available AlphaFold models (currently only available for *A. thaliana* proteins) (74) to the Phyre2-generated models showed good agreement in the inferred structures, confirming the presence of the cleavage sites in unstructured regions of the protein (AtEML2, AtKAN1, and AtGPI-AAL), although the exact positioning of intrinsically disordered regions of the proteins could not be modeled with precision with either program (Fig. 4 and 8 and Fig. S2 and S5), as already noted by others (75). In the case of AtPIF7, the cleavage site was located within a predicted α-helix in the AlphaFold model, although the confidence in the prediction of this region of the protein was only 70 to 90% (Fig. S8). Interestingly, the poorly processed AtFbKr and PrRHLP cleavage sites were located within predicted highly structured regions of the proteins (Fig. 8 and Fig. S9 and S10). In contrast, the AtPRC cleavage site, which was not cleaved *in vitro*, was not located near or within a predicted highly conserved structured domain. The Phyre2 model implied a weakly predicted α-helix in the region of the predicted cleavage site, which could have hindered its insertion in the protease substrate-binding pocket, although this prediction was not supported by the AlphaFold model (Fig. 8 and Fig. S11). Overall, modeling using Phyre2 or AlphaFold provided useful predictions on the presentation of the putative cleavage sites in the deduced protein structures, many of which were confirmed experimentally, although with a few exceptions. Thus, although modeling of protein structure still comes with limitations, it could be combined with the search for signature cleavage site sequences to improve the identification of the most promising candidate target proteins for downstream experimental validation.

We confirmed *in vivo* cleavage of ectopically expressed AtEML2, a nuclear histone reader protein (69–71). The protein was readily cleaved in TuMV-infected plants or in plants that coexpressed the TuMV or PPV proteases (Fig. 4 and 5). Since VPg-Pro and Pro are known to accumulate predominantly in the nucleus (35, 36, 38), we speculate that they are responsible for the cleavage observed in infected cells. A large proportion of candidate target proteins have confirmed or predicted nuclear localization (Table 3 and Excel Sheet S1), including several transcription factors. Other candidate proteins were predicted to localize to various compartments in the cell. The accessibility of these proteins to the NIa protease is not known. A subpopulation of VPg-Pro and Pro accumulates in the cytoplasm (35). In addition, the 6K-VPg-Pro is localized to ER membranes and may also localize with chloroplasts or other organelles at late stages of infection, together with the virus replication organelle (38, 41). We have shown partial *in vivo* cleavage of AtDUF707, a membrane protein, possibly localized in the mitochondria (Fig. 6). This result suggests that one or several forms of the NIa protease displays proteolytic activity that can target proteins outside the nucleus, although the exact subcellular localization of this particular cleavage event was not determined. Experimental validation of other candidate target proteins will be necessary to evaluate the activity of the NIa protease in various subcellular compartments.

We acknowledge that further experiments will be necessary to confirm cleavage of endogenous plant proteins by potyvirus NIa proteases and to evaluate the biological impact of these cleavage events. Many candidate target proteins are transcriptional or epigenetic regulators, some of which (including AtEML2, PpP100, and PpDDB) are known or predicted to play a role in orchestrating defense responses to pathogen attack (Fig. 2 and Tables 2 and 3 and Excel Sheet S1). Cleavage of some of these factors could hinder the mounting of the plant defense response or contribute to the massive reprogramming of the plant transcriptome observed in PPV- and TuMV-infected plants (76–80). EML proteins have been identified as key players of host defense responses to fungal and geminivirus infection (69, 71). Further work will be aimed at examining the biological consequence of AtEML2 cleavage, for example, how it may impact the expression of host defense genes or how it may affect the function of other EMLs or EML-interacting proteins, possibly through *trans*-dominant negative action of the cleaved fragments. Although a canonical cleavage site sequence was not identified in peach or N. benthamiana AtEML2 homologs, KGGGHQA (peach, e.g., Prupe.7G075700.2) and QSGGHQA (N. benthamiana, e.g., Niben101Scf03893g02006.1) sequences can be found in the flexible linker between the ENT and Agenet/Tudor domains of these proteins. It will be interesting to determine if these sequences, which include the conserved H at P2, Q at P1, A at P1′ (peach and N. benthamiana), and Q at P6 (only N. benthamiana) are cleaved by the PPV protease, even if only partially.

Using ectopically expressed plant proteins, we present evidence for the cleavage of plant proteins by potyvirus proteases. Although cleavage of the corresponding endogenous plant proteins still needs to be confirmed, it can be anticipated that these cleavage events could have a significant impact on the virus infection cycle and on the manifestation and/or intensity of virus-induced symptoms. As such, the proteolytic cleavage of plant proteins by viral proteases likely represents an additional layer of plant-virus interactions. Approximately 40% of plant viruses encode proteases (6, 7), many of which, at least those with *trans*-cleavage activity, could target plant proteins. It can be anticipated that the elucidation of viral protease cleavage sites in plant proteomes could lead to the design of new antiviral strategies, for example, strategies based on the precise editing of identified cleavage sites to prevent their recognition by viral proteases.

## MATERIALS AND METHODS

### Purification of recombinant PPV and TuMV proteases.

Purified TEV Pro was purchased from New England Biolabs (Canada). Previously, we purified active recombinant Pro from PPV strain M (isolate SK 68) (81). We used a similar strategy to purify recombinant NIa proteases from PPV strain D found in Ontario (isolate PPV-VPMV; GenBank accession no. KU948432.1) and from TuMV strain UK1, for which an infectious clone is available (pCambia.TuMV.GFP, GenBank accession number no. EF028235.1, kindly provided by Jean-François Laliberté, INRS-Institut Armand-Frappier, Laval, Canada, and Aiming Wang, London Research and Development Centre, Agriculture and Agri-Food Canada, London, Canada) (72). cDNA fragments corresponding to the coding regions for wild-type or mutated PPV Pro and VPg-Pro were synthesized *de novo* by GenScript (Piscataway, NJ) after optimization of codon usage for expression in E. coli. The coding region for TuMV Pro was amplified by PCR with primers TuMV7236F-NcoI and TuMV7960R-XhoI from pCambia.TuMV.GFP (see Table S4 in the supplemental material). All DNA fragments were cloned into the NcoI and XhoI restriction sites of protein expression vector pET21-d(+) (Novagen, Madison, WI). All proteases included a 6×His tag fused to their C terminus to facilitate protein purification. The DNA sequences of the expression constructs were confirmed by sequencing.

The constructs were transformed into E. coli Rosetta-gami B(DE3)pLysS competent cells (Novagen) for protein expression. Small-scale pilot experiments were used to optimize the solubility of the expressed recombinant proteins. Cells were initially grown at 37°C and 250 rpm in 5 mL of Luria-Bertani (LB) medium containing 50 μg/mL ampicillin, 15 μg/mL kanamycin, 12.5 μg/mL tetracycline, and 34 μg/mL chloramphenicol until the optical density at 600 nm (OD_600_) reached 1.0. Expression was induced by addition of isopropyl-β-d-thiogalactopyranoside (IPTG) at 0.1 or 0.5 mM, and cells were grown at 18, 25, or 30°C for 3 h. Cells were then collected by centrifugation (10,000 × *g* for 5 min) and lysed with 1× SDS-PAGE protein loading buffer (82). For large scale expression and to improve protein solubility, 1-L cultures were grown at 30°C and induced with 0.1 mM IPTG. Cells were collected by centrifugation (2,500 × *g* for 15 min).

Recombinant proteins were purified by Ni^2+^ affinity chromatography. Cell pellets were lysed in 50 mL of B-PER bacterial extraction reagent (Thermo Scientific) containing 5 μl Benzonase nuclease (Novagen) and incubated at room temperature for 40 min with gentle shaking. After centrifugation at 16,000 × *g* for 20 min at 4°C, the supernatant was applied on a column of HisPur Ni-nitrilotriacetic acid (NTA) resin (Thermo Scientific). The column was washed with washing buffer (50 mM sodium phosphate, 0.3 M NaCl, and 20 mM imidazole [pH 7.5]), and recombinant proteins were eluted with elution buffer (50 mM sodium phosphate,0.3 M NaCl and 250 mM imidazole [pH 7.5]). Purified proteins were stored in elution buffer supplemented with 50% glycerol at −20°C. Protein concentrations were determined in triplicate using the DC protein assay (Bio-Rad, Hercules, CA) and bovine serum albumin as the standard.

### *In vitro* cleavage assays using a fluorescence peptide substrate.

A fluorescence peptide substrate, MCA-Ser-Asn-Val-Val-Val-His-Gln-Ala-Asp-Glu-Arg-Glu-Dap(DNP)-NH_2_, was synthesized based on the PPV NIb-CP cleavage site sequence (SNVVVHQADERE) located between the NIb and CP protein domains of the polyprotein, and incorporated a fluorophore, 7-methoxycoumarin-4-yl)acetyl (MCA), at its N terminus and a quencher, 2,4-dinitrophenyl (DNP), at its C terminus (Synpeptide, Shanghai, China). For simplicity, we refer to this peptide as MCA-QA-DNP. Reactions were carried out at room temperature in microtiter plates using 50 nM purified protease and various concentrations of the substrate MCA-QA-DNP (0.25, 0.5, 0.75, 1.0, 1.5, 2, 3, 4, 5, 6, 8, 10, 12, 14, 16, 20, 24, and 28 μM) in the optimized assay buffer (50 mM Tris-HCl [pH 7.5], 150 mM NaCl, 1 mM EDTA, 1 mM dithiothreitol [DTT], and 0.1% Brij35). Fluorescence was measured using a SpectraMax Gemini EM microplate reader (Molecular Devices, CA, USA). The excitation and emission wavelengths were 325 nm and 392 nm, respectively. Nonlinear regression with the Michaelis-Menten model was used to derive *K_m_*. The *k*_cat_ value was calculated using the following equation: *k*_cat_ = *V*_max_/[*E*], where [E] is the enzyme concentration.

### *In vitro* cleavage assays using partial viral polyprotein precursors or selected plant proteins.

cDNA fragments corresponding to the coding regions for the PPV, TuMV, and TEV NIb-CP were amplified by PCR from full-length cDNA clones pPPV-VPM, pTuMV.GFP, and pTEV.GFP, respectively (pTEV.GFP was a generous gift from James C. Carrington, Donald Danforth Plant Sciences Center, St. Louis, MO) using primers listed in Table S4 and the Q5 high-fidelity (HF) DNA polymerase (New England Biolabs, USA). All amplified fragments were cloned into vector pCITE4a to produce protein expression constructs for *in vitro* translation. For host proteins, total RNA was isolated from *A. thaliana* cv. Columbia and peach leaves using a modified protocol and the Spectrum plant total RNA kit (Sigma) (83). cDNAs were generated by reverse transcription using SuperScript Vilo master mix (Thermo Fisher, USA). The cDNAs were used as the templates for PCR using the primers listed in Table S4 and the Q5 HF DNA polymerase to amplify the coding regions for the six *A. thaliana* proteins and five peach proteins. The primers included restriction sites for cloning into vector pCITE4a (Novagen, USA). All constructs were designed to include the predicted NIa protease cleavage site. In most cases, the entire protein coding region was included, but for two larger proteins (AtGPI-AALP and PpCESA2), we selected a subfragment of the protein. For most constructs, the amplified cDNA fragment was digested with the restriction enzymes MscI/XhoI, NdeI/XhoI, or MscI/EcoRI and cloned into the corresponding restriction enzyme sites of the pCITE4a polylinker. However, PpCESA2, PpRHLP, and TEV NIb-CP were cloned into pCITE4a using NEBuilder HiFi DNA assembly master mix (New England Biolabs, USA). Mutations of the glutamine at the P1 position of the cleavage sites (glutamine to alanine) of proteins AtKAN1 and AtDUF707 were produced using the Q5 site-directed mutagenesis kit (New England Biolabs, USA). The sequence of all expression constructs was confirmed by sequencing.

*In vitro* translation reactions were conducted as described previously (84) using the TnT Quick coupled transcription/translation rabbit reticulocyte system (Promega, USA) and the EasyTag Express S^35^ protein labeling mix, which is a mixture of S^35^-labeled methionine and cysteine (PerkinElmer, USA). Briefly, the translation reactions were allowed to proceed at 23°C for 2 h and were arrested by the addition of cold methionine and RNase. A 5-μl aliquot of the translation reaction mixture was incubated with 50 nM NIa proteases in the activity buffer (50 mM Tris-HCl [pH 7.5], 150 mM NaCl, 1 mM EDTA, 1 mM DTT, and 0.1% Brij35), and the reaction mixtures were left at 16°C for 16 h. Samples were separated by electrophoresis on 12% or 15% SDS-polyacrylamide gels (82) using a Mini-Protean II electrophoresis cell (Bio-Rad, Hercules, CA). Radiolabeled protein bands were visualized by exposure of the dried gels using a Cyclone Plus phosphorimager (PerkinElmer). All experiments were repeated at least three times with similar results, and a representative result is shown for each experiment.

### *In vivo* cleavage assays of selected plant proteins in virus-infected plants or upon coexpression of viral proteases.

Plasmid constructs to allow the expression of the AtEML2-s and AtDUF707 proteins *in planta* were designed so that the expressed proteins were fused to the Myc epitope tag (provided from the vector pSITE-MYC) at their N terminus and to the Flag epitope tag at their C terminus. PCR fragments were generated using the *in vitro* expression plasmid constructs described above as the templates, gateway-specific primers, and Phusion high-fidelity DNA polymerase (New England Biolabs, USA) (see Table S4 for the primer list). The PCR fragments were recombined into pDONR221 to produce entry clones according to the supplier’s instructions (Life Technologies, Thermo Fisher). The inserts were then introduced into the destination vector pSITE-Myc (kindly provided by the late Michael Goodin, University of Kentucky, Lexington, KY). Mutation Q^80^A of the AtEML2-s construct was generated by site-directed mutagenesis as described above. To obtain vectors allowing the expression of AtEML2-l *in vivo*, a fragment corresponding to the open reading frame of EML2-l with an Myc tag at its N terminus and a Flag tag at its C terminus was synthesized *de novo* by GenScript (USA). An AtEML2-l mutant fragment with the mutation HQ^86-87^AA was also synthesized. Additional synthesized fragments corresponded to the expected Myc-tagged N-terminal cleaved fragment (N-AtEML2-l) or Flag-tagged C-terminal cleaved fragment (C-AtEML2-l). All fragments were cloned into the NcoI/BamHI sites of the polylinker of pBBI525 (containing a duplicate 35S promoter and the Nos terminator) (85). The cassettes, which included the 35S promoter, open reading frame, and Nos terminator, were subcloned into the HindIII/EcoRI sites of the plant expression vector pBINplus polylinker.

To produce clones for expression of PPV Pro *in planta*, a fragment corresponding to the open reading frame was amplified by PCR from full-length cDNA clones pPPV-VPM using primers PPV6294F-NcoI and PPV7022R-XbaI (Table S4) and cloned into the NcoI/XbaI sites of the pBBI525 polylinker. The cassette, which included the35S promoter, PPV Pro, and Nos terminator, was subcloned into the HindIII/SmaI sites of the pBINplus polylinker. Fragments corresponding to the TuMV Pro and mutant Pro C^2267^A open reading frame were synthesized *de novo* by GenScript. These fragments were subcloned into the NcoI/BamHI sites of the pBBI525 polylinker. The cassette, including the 35S promoter and the Nos terminator, was subcloned into the HindIII/SmaI sites of the pBINplus polylinker.

The constructs described above were transformed into A. tumefaciens GV3101. A. tumefaciens cultures were grown overnight at 28°C with shaking at 250 rpm in Luria-Bertani medium (Miller’s) containing various antibiotics depending on the individual constructs. A 0.5-mL aliquot of the overnight culture was transferred to 50 mL of fresh medium containing 10 mM 2-morpholinoethanesulfonic acid (MES; pH 5.6), 40 μM acetosyringone, and various antibiotics, and again cultured overnight. The bacteria were pelleted by spinning the cultures for 6 min at 4,000 × *g* and resuspended in 50 mL of freshly prepared infiltration buffer (10 mM MgCl_2_ and 200 μm acetosyringone). The bacterial suspensions were kept at room temperature for at least 3 h without shaking. They were then diluted to the appropriate OD_600_ as indicated in the figure legends, followed by agroinfiltration into the underside of 4- to 5-week-old N. benthamiana leaves using a 1-mL needleless syringe. For coinfiltration experiments, equal volumes of A. tumefaciens transformed with the target protein constructs and of A. tumefaciens transformed with Pro constructs or with the infectious TuMV clone were mixed before infiltration. Infiltrated leaves were collected at 2 days postinfiltration. Plant protein extracts were prepared by grinding 0.1 g of leaves in 0.4 mL of 2× SDS gel-loading buffer and separated by SDS-PAGE as described above. Proteins were electroblotted to a polyvinylidene difluoride (PVDF) membrane (Roche, Canada) for immunoblot detection. Commercial monoclonal anti-Myc antibody (Santa Cruz Biotechnology) and monoclonal anti-Flag antibody (Sigma) were used as primary antibodies, and goat anti-mouse horseradish peroxidase (HRP)-conjugated antibody (Medicorp, USA) was used as a secondary antibody. The immunoblot membranes were developed using the ECL Clarity kit (Bio-Rad) and visualized using a ChemiDoc MP imaging system (Bio-Rad). All experiments were repeated at least three times with similar results, and a representative result is shown for each experiment.

### Bioinformatics.

To reinvestigate the cleavage site specificity of the PPV, TuMV, and TEV NIa proteases, the full polyprotein sequences for all available PPV, TuMV, and TEV isolates were downloaded from NCBI GenBank. Amino acid sequences were aligned using MEGA X (86).

To identify putative host proteins targeted by NIa proteases, reference proteomes were searched using a regular expression search, using the Linux tool *grep* with the search term corresponding to the PPV cleavage site consensus sequence [EQN].V.H[QE]/[STGA]. The reference proteomes used were uniprot-proteome_UP000006548 for *A. thaliana*, Prunus_persica_NCBIv2.pep.all for *P. persica*, and Niben101_annotation.proteins.wdesc for N. benthamiana. For each protein sequence, original annotations were complemented with GO terms using InterProScan v5.45. Further annotations were provided by The Arabidopsis Information Resource (TAIR) *A. thaliana* GO annotations (http://www.arabidopsis.org) downloaded in June 2021. For peach and N. benthamiana proteins, we used BLASTP (minimum *P* < 1E−5) and the TAIR *A. thaliana* protein database as a template to assign functional GOs. For GO enrichment analysis, the *P* value for each annotation was calculated using the BINGO algorithm (https://doi.org/10.1093/bioinformatics/bti551) and a hypergeometric distribution. The reported *P* values are the adjusted *P* values after a Benjamini-Hochberg false-discovery rate (FDR) correction (alpha = 0.01). To examine enriched biological processes that were shared among the plant hosts, we only considered enriched GOs with adjusted *P* values of less than 0.05 and GOs associated with a minimum of 3 proteins in each plant host.

Secondary structure models were generated using Phyre2 (http://www.sbg.bio.ic.ac.uk/phyre2/html/page.cgi?id=index) (87), PSIPRED (http://bioinf.cs.ucl.ac.uk/psipred/) (88), and PredictProtein (https://predictprotein.org/; RePROF method) (89) with default settings. Solvent accessibility was predicted using the RePROF method available on the PredictProtein portal. Predictions of disorder state were generated using Phyre2 and the Meta-Disorder method available on the PredictProtein portal. Three-dimensional (3D) structure models were generated by homology modeling using the intensive mode in Phyre2 with default settings (87). The Phyre2 models were compared to publicly available structure models produced using the deep-learning AlphaFold method (74) (https://www.alphafold.ebi.ac.uk/; models currently only available for *A. thaliana* proteins). Visual images of all 3D models, including coloring of relevant regions, were created using EZMol (http://www.sbg.bio.ic.ac.uk/ezmol/) (90). Subcellular localization of AtDUF707 was predicted using AtSubP (http://bioinfo3.noble.org/AtSubP/index.php?dowhat=AtSubP) (91), TargetP (http://www.cbs.dtu.dk/services/TargetP/) (92), Plant-mPLoc (http://www.csbio.sjtu.edu.cn/cgi-bin/PlantmPLoc.cgi) (93), and LocTree3 (https://rostlab.org/services/loctree3/) (94).
