# Supplementary material for: Proteolytic Processing of Plant Proteins by Potyvirus NIa Proteases
Source: J Virol. 2022 Jan 26;96(2):e01444-21. doi: 10.1128/JVI.01444-21 (PMC8791256; doi:10.1128/JVI.01444-21)
Supplement: Supplemental file 1 — Tables S1 to S4; Fig. S1 to S11. Download JVI.01444-21-s0002.pdf, PDF file, 1.4 MB [file jvi.01444-21-s0002.pdf]

Table S1 . Consensus cleavage site sequence recognized by the PPV NIa protease derived from analysis of seven polyprotein cleavage sites

**Cleavage sites**	**P6**	**P5**	**P4**	**P3**	**P2**	**P1**	**P1'**
**P3-6K1**	Q220*, S1	V221	V221	V207, L12	H221	Q221	S220, N1
**6K1-CI**	Q195, H22, R4	T114, A97, S8, P2	V221	H217, Q2, R2	H221	Q221	S217, N4
**CI-6K2**	E221	C221	V221	H220, Q1	H221	Q221	T210, S7, N4
**6K2-VPg**	E220, D1	E218, G3	V220, A1	V177, A24, T12, I8	H221	Q220, P1	G221
**VPg-Pro**	E163, G54, D1, V1, K1, R1	E221	V221	D189, G32	H221	E220, D1	S220, G1
**Pro-NIb**	E206, D14, G1	F185, T21, I11, V4	V221	Y219, H2	N114, T106, A1	Q221	S217, A4
**NIb-CP**	N220, D1	I130, V91	V221	V162, I40, T9, M9, A1	H221	Q221	A217, V2, T2
**Consensus sequence****	**E, q, n**	**e, v, c, f, t, i**	**V**	**v, h, y, d**	**H, n, t**	**Q, e**	**S, g, a, t**

* 221 full PPV genome sequences were used to assess the conserved sequence at the seven cleavage sites of the polyproteins that are processed by the NIa protease. Numbers indicate how many sequences have a specific amino acid at the corresponding position of the cleavage site (e.g. 220 of 221 sequences have Q in the P6 position of the P3-6K1 cleavage site).

** Amino acids in uppercase letters are predominant at this position for all or most cleavage sites, underlined amino acids are the most conserved at this position.

Table S2. Consensus cleavage site sequence recognized by the TuMV NIa protease derived from analysis of seven polyprotein cleavage sites

Cleavage sites	P6	P5	P4	P3	P2	P1	P1'
**P3-6K1**	K468*, R5, E4, Q1	A330, V91, E18, T13, S12, L8, P4, M1, I1	V478	V427, T16, I16, A18, G4, K4, E2, M1	H478	Q477, D1	A470,
**6K1-CI**	P478	T461, N10, A4, S3	V478	Y472, H6	H478	Q478	T375, A103
**CI-6K2**	E477, G1	A478	V478	H478	H478	Q478	N346, S132
**6K2-VPg**	E478	P474, S3, H1	V478	T379, V72, I21, A6	H478	E478	A476, S2
**VPg-Pro**	V342, I116, T10, A6	P446, S25, Q4, L3	V478	D476, G2	H478	E478	S478
**Pro-NIb**	T474, M4	A474, P2, V2	V478	Y478	A478	Q478	T476, M2
**NIb-CP**	V342, A110, M18, T7, I4, E1	C452, R21, G3, S1, F1	V475, A3	Y475, C3	H475, S3	Q475, R3	A477, T1
**Consensus sequence****	**e, p, v, k, t**	**a, p, t, c**	**<u>V</u>**	**Y, v, h, t, d**	**<u>H</u>, a**	**<u>Q</u>, e**	**A, t, n, s**

* 478 full TuMV genome sequences were used to assess the conserved sequence at the seven cleavage sites of the polyprotein that are processed by the NIa protease. Numbers indicate how many sequences have a specific amino acid at the corresponding position of the cleavage site (e.g., 468 of 478 sequences have a K in the P6 position of the P3-6K1 cleavage site).

**Amino acids in uppercase letter are predominant at this position for all or most cleavage sites, underlined amino acids are the most conserved at this position.

Table S3. Consensus cleavage site sequence recognized by the TEV NIa protease derived from analysis of seven polyprotein cleavage sites

**Cleavage sites**	**P6**	**P5**	**P4**	**P3**	**P2**	**P1**	**P1'**
**P3-6K1**	E13*	D13	L13	V10, I3	E13	Q13	A13
**6K1-CI**	E13	I10, V3	I12, V1	Y13	T13	Q13	S13
**CI-6K2**	E13	T13	I12, V1	Y13	L13	Q13	S13
**6K2-VPg**	E13	P13	V13	Y13	F13	Q13	G13
**VPg-Pro**	E13	D10, E3	L13	T12, M1	F13	E13	G13
**Pro-NIb**	E13	L13	V13	Y13	S13	Q13	G13
**NIb-CP**	E13	T6, N4, A3	L13	Y13	F13	Q13	S12, G1
**Consensus sequence****	**E**	**d, t, p, l, i**	**L, V, I**	**Y, v, t**	**F, e, l, s, t**	**Q, e**	**G, S, a**

* 13 full TEV genome sequences were used to assess the conserved sequence at the seven cleavage sites of the polyprotein that are processed by the NIa protease. Numbers indicate how many sequences have a specific amino acid at the corresponding position of the cleavage site (e.g., 13 of 13 sequences have a E in the P6 position of the P3-6K1 cleavage site).

**Amino acids in uppercase letter are predominant at this position for all or most cleavage sites, underlined amino acids are the most conserved at this position

Table S4. Primers used in this study

Genes	Primer name	Primer sequences (5' to 3')	Purpose
TuMV NIa-Pro	TuMV7236F-NcoI	TACCATGGCTAGTAACTCCATGTTCAGAGG	Cloning of cDNA fragment into the NcoI-XhoI sites of pET21d(+) for expression of recombinant protease
	TuMV7960R-XhoI	ATTACTCGAGTGCGTAGACTGCCGTGCTG	
PPV NIb-CP	PPV8019F-MscI	TTATTATGGCCAAGCTTGGATACCATCCGGATAC	Cloning of cDNA fragments into pCITE-4a for *in vitro* cleavage of partial viral polyproteins or putative target plant proteins. The cloning site into pCITE-4a were MscI and XhoI for PPV NIb-CP, TuMV NIb-CP, AtEML2, AtDUF707, AtGPI-AALP and PpSLK2 cDNA fragments; NdeI and XhoI for AtKAN1, AtPIF7, AtFbKr and AtPRC; MscI and EcoRI for PpDDB and PpP100. PpRHLP, PpCESA2 and TEV NIb-CP were cloned into pCITE4a using NEBuilder HiFi DNA Assembly Master Mix.
	PPV9566R-XhoI	TTATTACTCGAGCACTCCCCTCACACCGAG	
TuMV NIb-CP	TuMV8960F-MscI	TTATTATGGCCAAAAGCGGAATTCCAAGTGAG	
	TuMV10381-SacI	TTCGAGCTCTCACAACCCCTGAACGCCCA	
TEV NIb-CP	TEV7965F	ATATGGCCACCACCCATTACACATGTGAGAAGTGTG	
	TEV9305R	TGGTGGTGGTGGTGCTCACTGGCGGACCCCTAAT	
AtEML2	EML2-284F-MscI	TTATTATGGCCATGGAAGCGCAGATTCATATAC	
	EML2-1225R-XhoI	ATTACTCGAGTCACCCCAGCAGCATTGGAAG	
AtDUF707	DUF707-682F-MscI	TTATTATGGCCATGAAGATCATTGCAACGGCA	
	DUF707-1848R-XhoI	ATTACTCGAGTTAAATGGTTGTGGCTGTTG	
AtKAN1	KAN1-675F-NdeI	GTACATATGTCTATGGAAGGTGTTTTTC	
	KAN1-1886R-XhoI	ATTACTCGAGTCATTTCTCGTGCCAATCTG	
AtPIF7	AtPIF7-327F-NdeI	GTACATATGTCGAATTATGGAGTTAAAGAG	
	AtPIF7-1427R-XhoI	ATTACTCGAGCTAATCTCTTTTCTCATGATTCG	
AtGPI-AALP	GPI1386F-MscI	TTATTATGGCCGCAACAGGAACCAGCTACTG	
	GPI2429R-XhoI	ATTACTCGAGGGCTGCAGATGGCTTTGTAT	
AtFbKr	FbKr165F-NdeI	GTACATATGAAGAGATTACCTTTGCATC	
	FbKr1472R-SacI	TTCGAGCTCCAACTCTTTCTTCGCACCCTT	
AtPRC	PRC147F-NdeI	GTACATATGTGCAATTGCTCATCTTCCTTC	
	PRC1133R-XhoI	ATTACTCGAGCTAGCGGAATATGTCCCATTCT	
PpDDB	PpDDB678F-MscI	TTATTATGGCCATGGATTCTGGTAGTGGAAG	
	PpDDB2087R-EcoRI	CAGAATTCCTAATCTGAGGAGCAAATCC	
PpSLK2	PpSLK2-474F-MscI	TTATTATGGCCATGCCGCCTAAAAGGAAGCAA	
	PpSLK2-2447R-XhoI	ATTACTCGAGTCAGACCTTCCAACCATAAC	
PpP100	PpP100-194F-MscI	TTATTATGGCCATGATCGTCATCAGCAGAGCAG	
	PpP100-1561R-EcoRI	CAGAATTCTTAGCACCACTCCCCATATAAAAC	
PpCESA2	CSAs2-946F	ATATGGCCACCACCCATATGGATACCAAAGGAAGACTTG	
	CSAs2-2307R	TGGTGGTGGTGGTGCCTATACTTTGTCTCTCAAATAGTC	
PpRHLP	PpRHLP1F	ATATGGCCACCACCCATATGACGGTACCTAAGACGGTC	
	PpRHIP1452R	TGGTGGTGGTGGTGCTCAGTGTGGAGCGATGGCCA	
AtKAN1	KAN1QA-F	GTTCATCATGCATCATCGACG	Mutation of the P1 residue in the cleavage site (Q to A mutation)
	KAN1QA-R	TTCGTTTCCATTTATGCCCA	
AtDUF707	DUF-QA-F	GTCGTTCATGCATCTTTTCCTTC	
	DUF-QA-R	CCATTGAGAATCAACTACTCC	
AtEML2-s	EML2QA-F	GTTAGACATGCGTCCCTCGATG	
	EML2QA-R	AACCTGGTTTCCACCTCTTGG	
AtEML2-s	attB1-EML2-F284	GGGGACAAGTTTGTACAAAAAAGCAGGCTTCATGGAAGCG<br>CAGATTCATATAC	Gateway cloning to pSITE-MYC vector
	attB2-EML2-R1225-flag	GGGGACCACTTTGTACAAGAAAGCTGGTGCTTA<br>CTTGTCGTCATCGTCTTTGTAGTCCCCCAGCAGCATTGGAAG	
AtDUF707	attB1-DUF-F682	GGGGACAAGTTTGTACAAAAAAGCAGGCTTCATGAAGATCA<br>TTGCAACGGCA	
	attB2-DUF-R1848-flag	GGGGACCACTTTGTACAAGAAAGCTGGTGCTTA<br>CTTGTCGTCATCGTCTTTGTAGTCAATGGTTGTGGCTGTTG	
PPV NIa-Pro	PPV6294F-NcoI	TGTCCATGGCTAGTAAATCACTGTTCAGAGG	Cloning into vector pBBI5252 and pBINplus
	PPV7022R-	ATTCTAGACTAGTGGTGGTGGTGGTGGTGCTGAGTGTAAAC<br>AAATTCC	

A

>PPV VPg-Pro-NIb-CP partial polyprotein

GFNRRQRQKLKFRQARDNRMAREVYGDDSTMEDYFGSAYSKKGKSKGKTRGMGTKTRKFVNMYGYDPTDYNFVRFVDPLTGHTLDENPLMD
INLVQEHFSQIRNDYIGDDKITMQHIMSNPGIVAYYIKDATQKALKVDLTPHNPLRVCDKTATIAGFPEREFELRQTGHPVFVEPNAIPKI
NEEGDEEVDHESKSLFRGLRDYNPIASSICQLNNSSGARQSEMFGLGFGGLIVTNQHLFKRNDGELTIRSHHGEFVVKDTKTLKLLPCKGR
DIVIIRLPKDFPPFPKRLQFRTPTTEDRVCLIGSNFQTKSISSTMSETSATYPVDNSHFWKHWISTKDGHCGLPIVSTRDGSILGLHSLAN
STNTQNFYAAFPDNFETTYLSNQDNDNWIKQWRYNPDEVCWGSLQLKRDIPQSPFTICKLLTDLDGEFVYTQSKTTHWLRDKLEGNLKAVG
ACPGQLVTKHVVKGKCTLFETYLLTHPEEHEFFRPLMGAYQKSALNKDAYVKDLMKYSKPIVVGAVDCDQFERAVDVVISMLISKGFEECN
YVTDPDDIFSALNMKAAVGALYSGKKRDYFKNVSDQDKESFVRASCKRLFMGKKGVWNGSLKAELRPKEKVEANKTRSFTAAPIDTLLGGK
VCVDDFNNQFYSLNLHCPWSVGMTKFRGGWDKLLRALPEGWIYCDADGSQFDSSLSPYLINAVLNIRLAFMEEWDIGEQMLSNLYTEIVYT
PIATPDGTIVKKFKGNNSGQPSTVVDNTLMVILAMTYSLLKLGYHPDTHDCICRYFVNGDDLVLAVHPAYESIYDELQEHFSQLGLNYTFA
TKTENKEELWFMSHKGVLYDDMYIPKLEPERIVSILEWDRSNEPIHRLEAICASMVEAWGYKELLREIRKFYSWVLEQAPYNALSKDGKAP
YIAETALKKLYTDTEASETEIERYLEAFYDDINDDGESNVVVHQADEREDEEEVDAGKPIVVTAPAATSPILQPPPVIQPAPRTTAPMFNP
IFTPATTQPATKPVPQVSGPQLQTFGTYGNEDASPSNSNALVNTNRDRDVDAGSIGTFTVPRLKAMTSKLSLPKVKGKAIMNLNHLAHYSP
AQVDLSNTRAPQSCFQTWYEGVKRDYDVTDDEMSIILNGLMVWCIENGTSPNINGMWVMMDGETQVEYPIKPLLDHAKPTFRQIMAHFSNV
AEAYIEKRNYEKAYMPRYGIQRNLTDYSLARYAFDFYEMTSTTPVRAREAHIQMKAAALRNVQNRLFGLDGNVGTQEEDTERHTAGDVNRN
MHNLLGVRGV

B

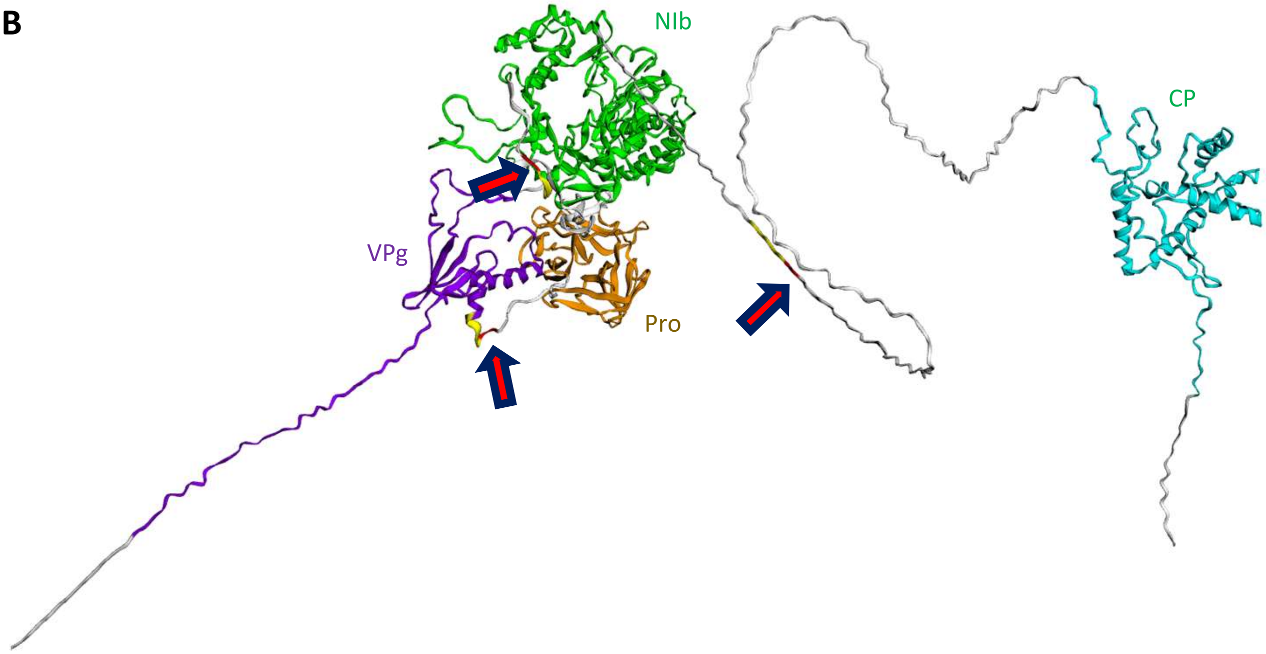

Figure S1. Structural model of the partial viral polyprotein including the NIa-VPg, NIa-Pro, NIb and CP domains (A) Sequence of the partial polyprotein. Regions of the polyprotein with 3D structure predicted with a high degree of confidence are underlined (VPg in purple, Pro in brown, NIb in green and CP in blue). Letters highlighted in yellow correspond to the P6 to P1' positions of the predicted cleavage sites with the P1 and P1' positions underlined in red. (**B**) Structural model of the partial polyprotein predicted using Phyre2 (see Materials and Methods). The degree of confidence in the model varied with the region of the polyprotein. The VPg domain (highlighted in purple) was modeled with a very high degree of confidence (100%) based in part on the solved structure of the VPg from potato virus Y (pdb: 6NFW). The Pro and NIb domains (highlighted in brown and green, respectively) were also modeled with a very high degree of confidence (100%) based in part on the solved structure of the 3CD (Pro-Pol) of poliovirus (pdb: 2IJD). The CP domain (highlighted in brown) was modeled with a medium degree of confidence (74%) based in part on the solved structure of the CP from turnip mosaic virus (pdb: 6T34). The position of the predicted cleavage sites (P6 to P2 position highlighted in yellow; P1 and P1' positions highlighted in red) are shown with the red arrow. They are contained in flexible region of the polyprotein linking the different domains. Please note that the exact positioning of these linkers within the 3D structure of the protein could only be modeled *ab silico* with a low degree of confidence.

A >AtKan1

```
MSMEGVFLEKTKTNTTTTLPDLSLHISLPDIHQYHHNESSKESSRRSSQLENNNRSSNFELSLSHHNHPTARIFHCPDRR
TLNLPHQQHYNNPIINGVHQRVDESEISNLHRPIRGIPVYHNRSFPFHQQNSSLPSLGGGDMDQISILNSSSGYNNAYRS
LQSSPRLKGVPLHHHHHHNQYGVVGSSDSSSPHHHNHHHHGMIRSRFLPKMPTKRSMRAPRMRWTSSLHARFVHAVELLG
GHERATPKSVLELMDVKDLTLAHVKSHLQMYRTVKTTNKPAASSDGSGEEEMGINGNEVHHQSSTDQRAQSDDTSLHQET
DISSTQPRWSNSSRETWPLSNNCSSDIDTMIRTSSTSMISHYQRSSIQNQEQRSNDQAKRCGNLSCENPSLEFTLGRPDW
HEK
```

B **C** **D**

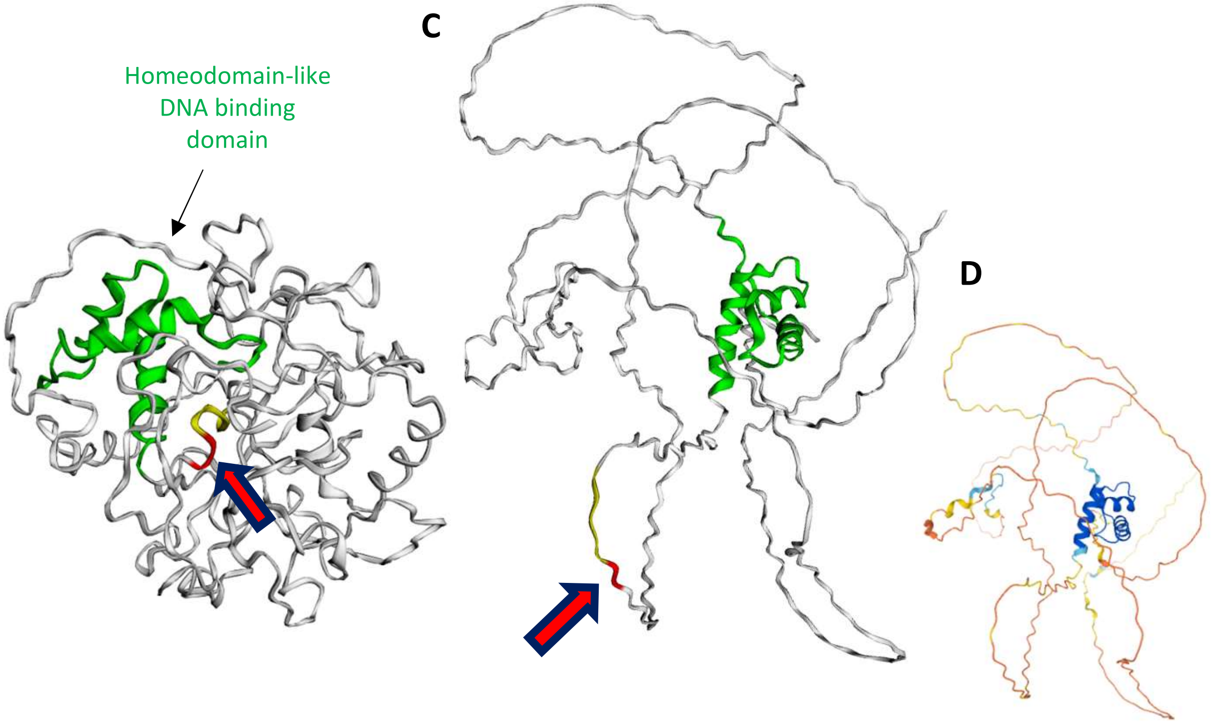

Figure S2. Structural models of the AtKan1 protein **(A)** Sequence of AtKan1. A predicted homeodomain-like DNA binding domain is underlined in green. Letters highlighted in yellow correspond to the P6 to P1' positions of the predicted cleavage site with the P1 and P1' positions underlined in red. (**B-D**) Structural model of AtKan1 predicted using Phyre2 (**B**, see Materials and Methods) or Alphafold (**C**, PDB file of the model available at https://www.alphafold.ebi.ac.uk/). For both models, the color scheme is as follows: homeodomain-like DNA binding domain in green, P6 to P2 positions of the predicted cleavage site in yellow and P1 to P1' position of the cleavage site in red. The position of the cleavage site is highlighted with the red arrow. The degree of confidence in the Phyre 2 model **(B)** varied with the domains of the protein. The predicted homeodomain-like DNA binding domain was modeled with a very high degree of confidence (99.9%) based in part on the solved structure of a similar domain from the myb2 domain of phosphate starvation response regulator 1 (pdb: 6J5B). Other regions of the protein were predicted to be mostly disorganized and the 3D structure of these regions could only be modeled *ab silico* with a low degree of confidence. The degree of confidence in the Alphafold model **(C)** also varied with the region of the protein, as shown in **(D)** and using the following color scheme: dark blue (more than 90 %), light blue (70-90 %), yellow (50-70%) and orange (less than 50%). In both models, the cleavage site is in a region of the protein predicted to be disorganized.

A

>PpP100

MIVISRAAEDDLSVPVGFRFHPTDEELVTHYLKKKLKGMDSHVSNIIREIDILKFEPWDLPERSLLKSDDENWFFFSRPEYNKHK
KNRTTQEGFWKITGREHQIKARDSRSVIGRKRILTFYRGRVRSSERTNWVMHEYYIPNDNPNAQRDFVLCRLKKNVKKSDENADV
AATCDEGETHNASDVENQQVNDMNMEDNRPPENLDYFERERDRLLANSLSNNDHNAFPTEFSANDQEFLRTLIVEPQSRETSDTD
REPVYHQSLQMRCEPQIPYELLQYGSSQSRRDTDVILHNQLSRQASSSVNVASKAGTYQREHRPQQQSGPIIVFRDTSADEYYTR
EKTRRITYPPEKPKEPEKPKPKPEKPKEPPYPRTAADFPPKQISITKSSIDKKVPQGSMEQTQNRTTPRNWKGSFITWQTSPLTS
PPSVYIFNTVLGAILFLFCVREVVLYGEWC

B

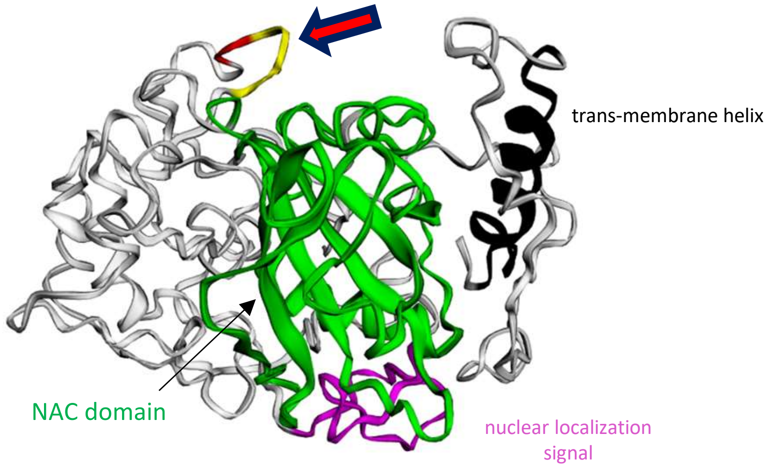

C

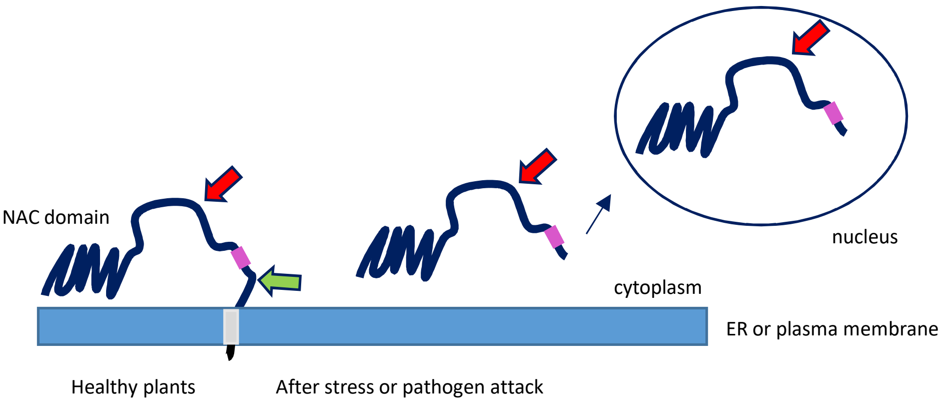

Figure S3. Structural model of the PpP100 protein (A) Sequence of PpP100. A predicted NAC domain is underlined in green. Predicted nuclear localization signal and trans-membrane helix are highlighted in pink and grey, respectively. Letters highlighted in yellow correspond to the P6 to P1' positions of the predicted cleavage site with the P1 and P1' positions underlined in red. (**B**) Structural model of Pp100 predicted using Phyre2 (see Materials and Methods). The degree of confidence in the model varied with the region of the protein. A predicted NAC domain (highlighted in green) was modeled with a very high degree of confidence (100%) based in part on the solved structure of the stress-induced transcription factor nac1 of rice (pdb: 3ULX). With the exception of the putative trans-membrane helix, other regions of the protein (including the putative nuclear localization signal in pink) were predicted to be mostly disorganized and the 3D structure of these regions could only be modeled *ab silico* with a low degree of confidence. The position of the predicted cleavage site (P6 to P2 position highlighted in yellow; P1 and P1' positions highlighted in red) is shown with the red arrow. The cleavage site is in a region of the protein predicted to be disorganized. **(C)** Topology model of the PpP100 protein in association with the plasma or ER membrane. A putative trans-membrane helix is shown in grey and a putative nuclear localization signal is shown in pink. The protein is predicted to be anchored to the membrane under normal conditions. Cleavage by a plant protease (shown with the green arrow) under stress conditions (including pathogen attack) would release the protein which would then be translocated into the nucleus. The putative NIa protease cleavage site is located between the NAC domain and the nuclear localization signal and may prevent translocation to the nucleus. Alternatively, cleavage of PpP100 could also occur in the nucleus.

A

>PpDDB

```
MDSGSGSMQSSSGGDDEYDSRAESISALLSNPPSQLGHMSSHAPPHHHHHHHHQQTHHHLDPLSNMFDPLSSRLTNPNP
LLNFDMAWSKTLRSDPNPTDLGGLSQPFLTNPNINQLGQSRGGGGGGGGSSTFAALQIPHDHQNVSASSSAPNNQTHNI
NSNSNNNNSNSNGVVRNPKKRSRASRRAPTTVLTTDTTNFRAMVQEFTGIPAPPFTSSSPFPRSRLDLFSSAAAASALM
RSAGGGGGGGGGGGLGLEPSPPSYLLRPFAHKVSHQPPSSSSILDHPNLPSTNSSATNHHNNLLNMQQNPSPSSSAVLN
FQSLFQPQHQQQQPKYSLPINSPNDLLASKTPHHHHHQGSLDHFGLTQQQLNVLPNNIVSSSDAALSRHDSNSNWGNGT
GPSNNNKTNIDNNNVDHQGLMRSINGNYGNGKLNYSAGSSSNNIIHGDKAQDQNVAAAAARSEGMVESWICSSD
```

B

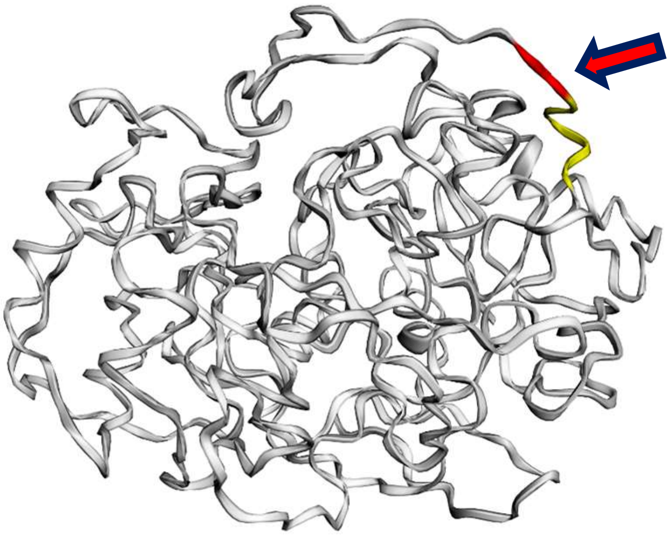

Figure S4. Structural model of the PpDDB protein. (A) Sequence of PpDDB. Letters highlighted in yellow correspond to the P6 to P1' positions of the predicted cleavage site with the P1 and P1' positions underlined in red. (**B**) Structural model of PDDB predicted using Phyre2 (see Material and Methods). This protein is predicted to be highly disorganized and its 3D structure could only be modeled *ab silico* with a very low degree of confidence. The position of the predicted cleavage site (P6 to P2 position highlighted in yellow; P1 and P1' positions highlighted in red) is shown with the red arrow.

A >AtGPI-AAL

MRPAIPLDYAVFQLSPKRSRCELFVSTTGNTEKLASGLVKPFVAHLKVAEEQVSREVQSIRLEVESNKNAGTWFTKGTLERFVRFVSTPEV
LELVSALDVEMSQLEAARKIYGEGTSDQRSSAKDSTDTTPAADVTKKELLKAIDLRLAAVRQDLATACNRASAAGFNPITVSELSQFADRF
GANRLNEACTKFITLCQRRPELMSSWRVNQEEEAIRSSWESDMSIDDPSEDPSRDLATNRNQQHREYQTGMEEQSATGTSYCQHESKLKPQ
SSHDENDEEEEKSTVQNEPLVSQPRQLTRRLSVQERISMFENKQKENSGEKTAVAKSTELKRLSSDLSSSAGMEKVVVRRWSGASDMSIDL
GNDRKDDTGDSPLCTPSSSSVSKDGSGASSKQFVGYNKKEQNGLSHAANPHRNEEECTSNNGGDWGMDEVESQNSSSTFLPKDKEVDLNVP
FRTNNQVRHQGNSPDRYLEKNSKYKFHEKNPRASSDYTGNANINDDANNQMSDFISNRQNQIQFRDPQSHSLSTLQQLGGTEPIITSVQSN
GVTAESPRKELMPSDRQSPLLEDRQRKTPFSGGSEQMKRPHSRRPEMGSAAVNTKPSAAINSVSDISESDTLIQVSPTEQVQRARPSKGSQ
ELNDELKVKANELEKLFAEHMLRVPGDQSSSVRRGKPGKPSEQAVTSQLRRPVAQDLSSVQISDQKTLAMPTLTSNDEDKFKTPPTMKMVV
TKDYGDTTRQNFPEISFSDNSRGKFYEQYMQKRDAKLKEDWSCRRTEKEAKLKVMQDILDRSNAEMKTKFSQSTGRRDSSARRAEKLVYFN
SKLSAKKDQHPISSFQSEEDEDGSRSTQNKKLQQNKNNLLIARTTATSASRSAAKVSTLSAVRRRGQSEKHFAQSVPNFSEIKKEGMKPAS
GVGKNGVRTQVRSSIRPKAVNEEEKLRRPKIFRKGAAEAAELATDFSQLKSEDGVSVPLYLEQEQSGRNFNSHGTGISSDNAQLKASEESE
ASDDMEKEGMGEALDDTEVEAFTDAENEMPRLSQESEEWGSTGVANGESFSQLDAGSNTELPAAMASRHQTMGSILDSPGESTSPWNSRVK
HRYPNEASELDASVDSPVGSPAFWNFSSLNHTESDTTQMRKKWGAAQKRAAGGNPSQNQCQQDVTKGLKRLLNFGRKNRAAESLADWISAT
TSEGDDDTDDGRDLANRSSEDLRKSRMGFLQSHPSGDSFNESELFTEHVQTTGTPLSFKLKEDQTTGASVKAPRSFFSLSNFRSKGK

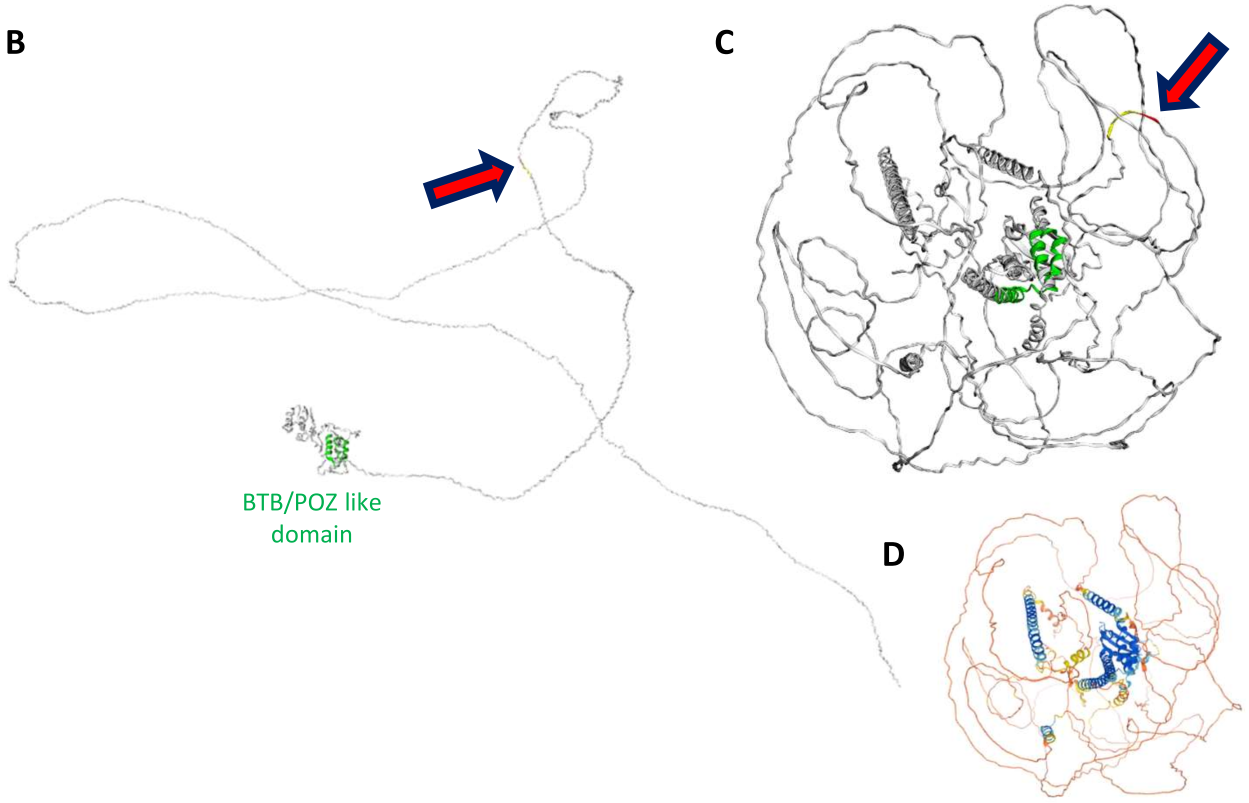

Figure S5. Structural models of the AtGPI-AAL protein (A) Sequence of AtGPI-AAL. A predicted BTB/POZ-like domain is underlined in green. Letters highlighted in yellow correspond to the P6 to P1' positions of the predicted cleavage site with the P1 and P1' positions underlined in red. (**B-D**) Structural models of AtGPI-AAL predicted using Phyre2 (**B**, see Materials and Methods) or Alphafold (**C**, PDB file of the model available at https://www.alphafold.ebi.ac.uk/). For both models, the color scheme is as follows: BTB/POZ like domain in green, P6 to P2 positions of the predicted cleavage site in yellow and P1 to P1' position of the cleavage site in red. The position of the cleavage site is highlighted with the red arrow. The degree of confidence in the Phyre 2 model **(B)** varied with the domains of the protein. The predicted BTB/POZ-like domain (in green) was modeled with a very high degree of confidence (94.8%) based in part on the solved structure of the human speckle-type poz protein btb domain (pdb: 4J8Z). Other regions of the protein were predicted to be mostly disorganized and the 3D structure of these regions could only be modeled *ab silico* with a low degree of confidence. The degree of confidence in the Alphafold model **(C)** also varied with the region of the protein, as shown in **(D)** and using the following color scheme: dark blue (more than 90 %), light blue (70-90 %), yellow (50-70%) and orange (less than 50%). In both models, the cleavage site is in a region of the protein predicted to be disorganized.

A >PpSLK2

MPPKRKQYQWHFGAAPQPALKNHHSLLNGGEQEPLTSSQRHNKPRIDVKKEASLNKHAIQQLLQSQDSEELQRNKLQIQELFH
YNMSQNQDQPKILHPSLQLKGDDKEKQQQPMRHVVTQQEVVHQASVMQLPDEGVCSRRLMQYIYHLRNRPADNNLSYWRKFVA
EYYAPSAKKRWCLSSYDEVGRDALGILPHLTMVPWQCNICGCKSRRGFEAYFEVLPRLNEITFGSGVIDELLFLDLPREIRFP
SGVMMLEYGRAVQESVYQQLHVVHEGQLRIVFSHDLKILSWEFCVCSHEVFFRRTAVAPQVVQLVHAVQDYKCSIDDRGSDGV
LFQDVQANCNRILAAGGQLAKTVDQQLVDDLGFSKRYTRCLQIAEIVYTMKDLMILCQDNVTGPIESLESYCRGAAMTKLQKQ
EIKGKEQLESARDPPKDNNKLMAASCGFRSNTNESSPMSHKGLSTSAELAASLLRGSHHKLMGQSNLTSIVSRASQEPHIQDT
SSEPFQGPRTSNPGLIKSSVENGLSSLDSSMKQYAIQKLVQEMINNNSRSANKHDREEPIWGSGKGSVIELPSGVWGCPTAAA
AQGNVFNSIAGRTSSSKAAFNGNSSEVHTNNCFINGEPNLSGKLCLPESIVNISHGYHDHNSIYGNGNDVGYGWKV

B

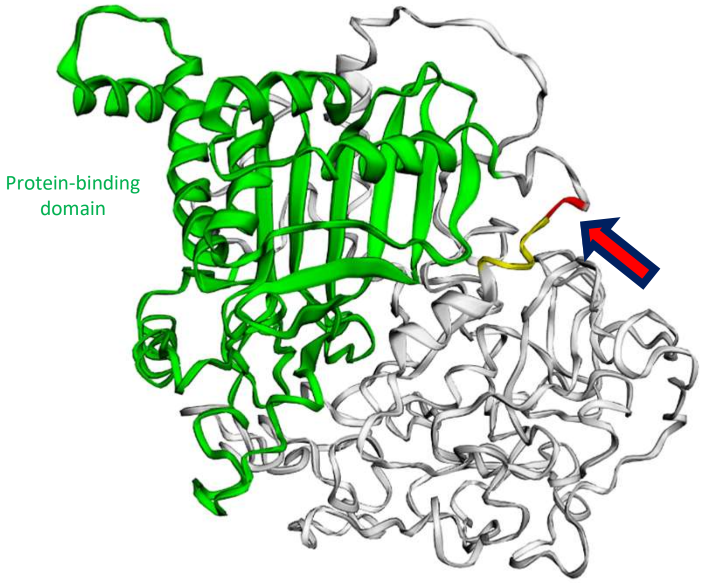

Figure S6. Structural model of the PpSLK2 protein (A) Sequence of PpSLK2. A predicted protein-binding domain is underlined in green. Letters highlighted in yellow correspond to the P6 to P1' positions of the predicted cleavage site with the P1 and P1' positions underlined in red. **(B)** Structural model of PpSLK2 predicted using Phyre2 (see Material and Methods). The degree of confidence in the model varied with the region of the protein. A predicted protein-binding domain (in green) was modeled with a very high degree of confidence (100%) based in part on the solved structure of the human ldb1 protein in complex with ssbp2 (pdb: 6TYD). Other regions of the protein were predicted to be mostly disorganized and the 3D structure of these regions could only be modeled *ab silico* with a low degree of confidence. The position of the predicted cleavage site (P6 to P2 position highlighted in yellow; P1 and P1' positions highlighted in red) is shown with the red arrow. The cleavage site is in a region of the protein predicted to be disorganized.

A >PpCESA2

MDTKGRLVAGSHNRNEFVLINADEVSRVTSVKELSGQICQICGDEIEITVDGEPFVACNECAFPVCRSCYEYERREGNQACPQCKTRYKRLKGSPRVEG
DEEEDDIDDLENEFDISSNDRRDPHHIAEAVLAARLNIGRGSHVHGSGISTPAEFDSASIASEIPLLTYGQEDVGIASDKHALIIPPFMSRGKRVHPMP
TTDSSMSFPPRPMDPKKDLAVYGYGTVAWKERMEDWKKKQNEKLQVVKHQGGNDGGNNNGNEPDDPDLPKMDEGRQPLSRKLPIPSSKINPYRMIILLR
LAILGLFFHYRILHPVNNAYGLWLTSIICEIWFGLSWILDQFPKWYPIERETYLDRLSLRYEKEGKPSELADLDVFVSTVDPLKEPPLITANTVLSILS
VDYPVDKVACYVSDDGAAMLTFEALSETSEFARKWVPFCKKYSIEPRAPEWYFAQKVDYLRDKVDPTFVRERRAIKREYEEFKVRINGLVATAQKVPEE
GWTMQDGTPWPGNNVRDHPGMIQVFLGQNGVRDVEGNELPRLVYVSREKRPGFDHHKKAGAMNSLVRVSAIISNAPYILNVDCDHYINNSRALREAMCF
MMDPTSGKKICYVQFPQRFDGIDRHDRYSNRNVVFFDINMKGLDGIQGPIYVGTGCVFRRQALYGYDAPTKKKPPGKTCNCLPKWCCWCCGSRKKNKKA
KSNDKKKKNKDASKQIHALENIQEGIEGIDNEKSSLIPQIKFEKKFGQSPVFIASTLMEDGGVPKGTSSASLLKEAIHVISCGYEDKTEWGKEVGWIYG
SVTEDILTGFKMHCHGWRSVYCMPKRPAFKGSAPINLSDRLHQVLRWALGSVEILLSRHCPIWYGYGCGLKWLERFSYINSVVYPLTSIPLLAYCSLPA
VCLLTGKFIVPEISNYASILFMALFLSIAATSILEMQWGHVGIHDWWRNEQFWVIGGASSHFFALIQGLLKVLGGVNTNFTVTSKAADDGEFSDLYLFK
WTSLLIPPMTLLIINIIGVVVGISDAINNGYDSWGPLFGRLFFAIWVIVHLYPFLKGLVGRQERLPTIIVVWSILLASIFSLLWVRINPFVSKGGIVLE
VCGLDCD

B

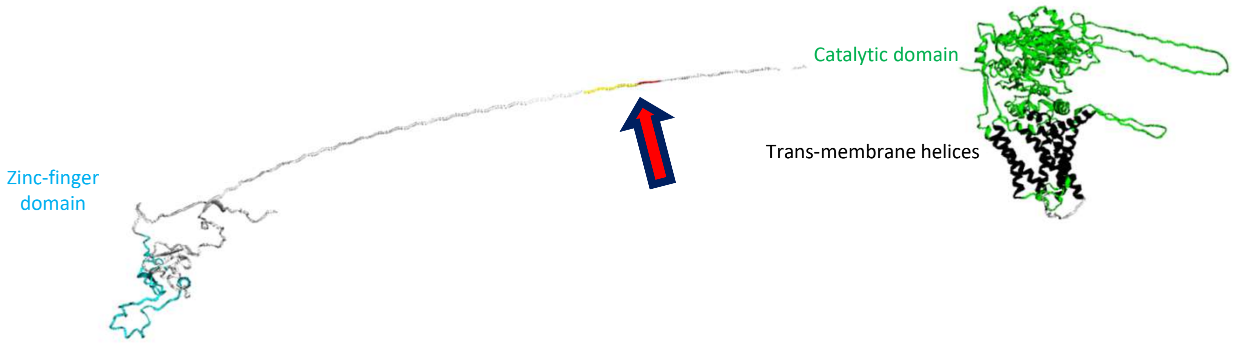

C

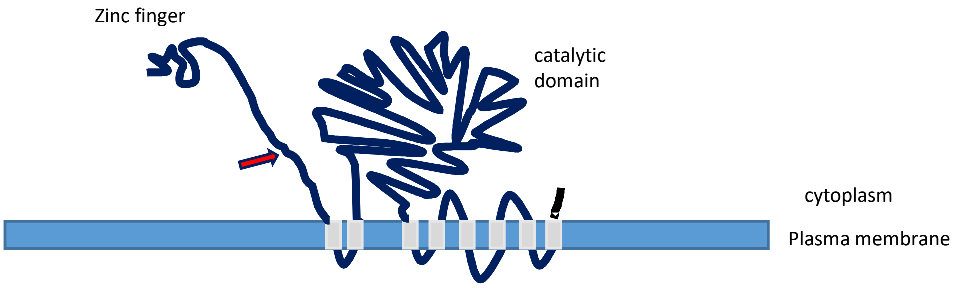

Figure S7. Structural model of the PpCESA2 protein (A) Sequence of PpCESA2. Predicted zinc-finger domain and catalytic domain are underlined in blue and green, respectively. Grey shadings correspond to predicted trans-membrane helices. Letters highlighted in yellow correspond to the P6 to P1' positions of the predicted cleavage site with the P1 and P1' positions underlined in red. **(B)** Structural model of PpCESA2 predicted using Phyre2 (see Material and Methods). In this model, 74% of the residues were modeled at a very high degree of confidence (100 %) based in part on the solved structure of the homotrimeric poplar cellulose synthase isoform 8 (pdb: 6WLB). Regions predicted with this high degree of confidence include the zinc-finger domain (in blue), the catalytic domain (in green) and 8 transmembrane helices (in black) . The position of the predicted cleavage site (P6 to P2 position highlighted in yellow; P1 and P1' positions highlighted in red) is shown with the red arrow. The cleavage site is in a flexible linker between the zinc-finger domain and the catalytic domain. **(C)** Topology model of the PpCESA2 protein in association with the plasma membrane. Trans-membrane helices are shown in grey. In this model, large portions of the protein (including the predicted cleavage site) are predicted to be on the cytoplasmic face of the membrane.

A >AtPIF7

MSNYGVKELTWENGQLTVHGLGDEVEPTTSNNPIWTQSLNGCETLESVVHQAALQQPSKFQLQSPNGPNHNYESKDGSCSRKR
GYPQEMDRWFAVQEESHRVGHSVTASASGTNMSWASFESGRSLKTARTGDRDYFRSGSETQDTEGDEQETRGEAGRSNGRRGR
AAAIHNESERRRRDRINQRMRTLQKLLPTASKADKVSILDDVIEHLKQLQAQVQFMSLRANLPQQMMIPQLPPPQSVLSIQHQ
QQQQQQQQQQQQQQQQFQMSLLATMARMGMGGGGNGYGGLVPPPPPPPMMVPPMGNRDCTNGSSATLSDPYSAFFAQTMNMDL
YNKMAAAIYRQQSDQTTKVNIGMPSSSSNHEKRD

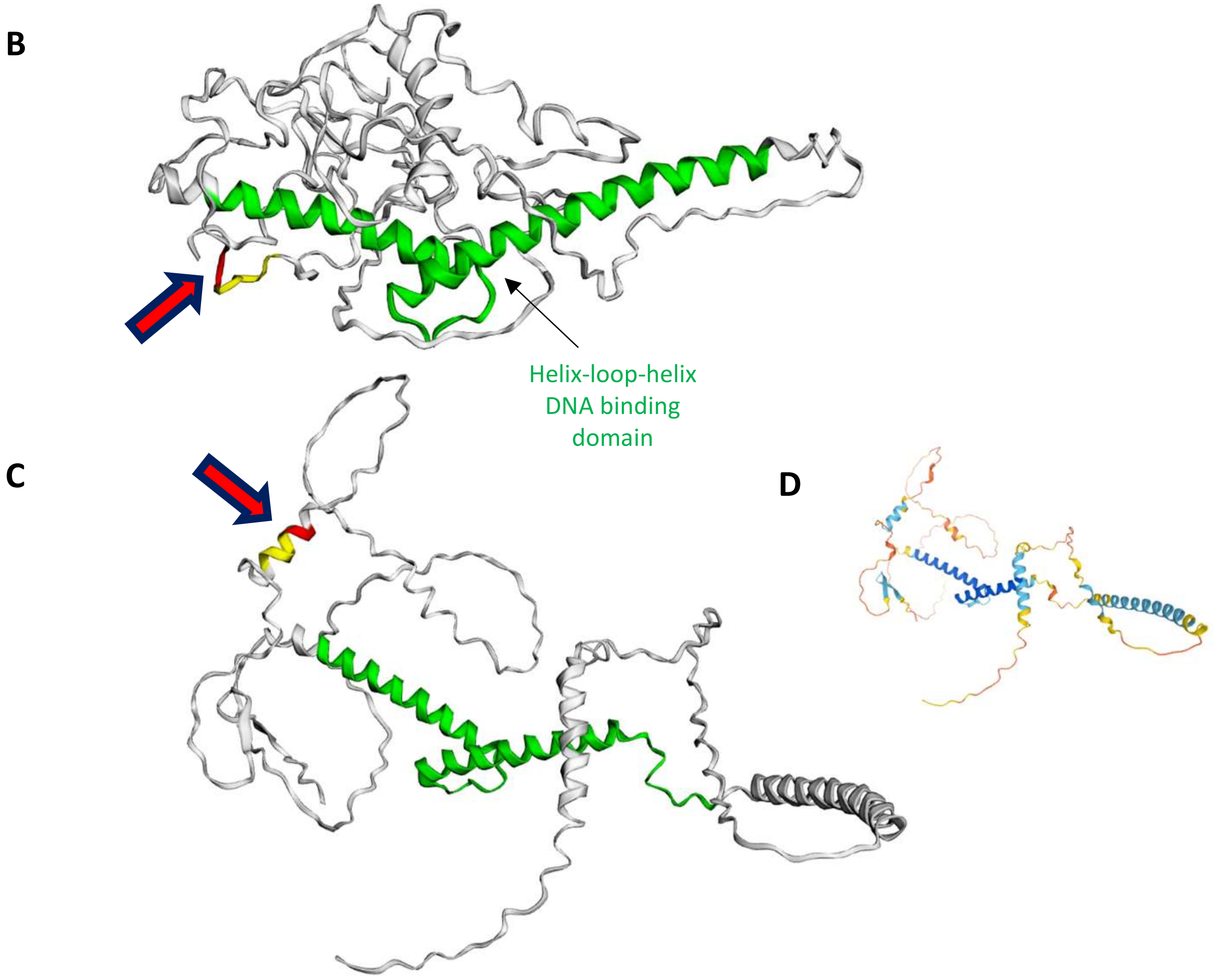

Figure S8. Structural models of the AtPIF7 protein (A) Sequence of AtPIF7. A predicted helix-loop-helix DNA binding domain is underlined in green. Letters highlighted in yellow correspond to the P6 to P1' positions of the predicted cleavage site with the P1 and P1' positions underlined in red. (**B-D**) Structural models of AtPIF7 predicted using Phyre2 (**B**, see Materials and Methods) or Alphafold (**C**, PDB file of the model available at https://www.alphafold.ebi.ac.uk/). For both models, the color scheme is as follows: helix-loop-helix DNA binding domain in green, P6 to P2 positions of the predicted cleavage site in yellow and P1 to P1' position of the cleavage site in red. The position of the cleavage site is highlighted with the red arrow. The degree of confidence in the Phyre 2 model **(B)** varied with the domains of the protein. The predicted helix-loop-helix DNA binding domain was modeled with a very high degree of confidence (99.7%) based in part on the solved structure of a similar domain from transcription factor myc2 (pdb: 5GNJ). Other regions of the protein were predicted to be mostly disorganized and the 3D structure of these regions could only be modeled *ab silico* with a low degree of confidence. The degree of confidence in the Alphafold model **(C)** also varied with the region of the protein, as shown in **(D)** and using the following color scheme: dark blue (more than 90 %), light blue (70-90 %), yellow (50-70%) and orange (less than 50%). The positioning of the cleavage site differed in the two models. It was located on a flexible region of the protein in the Phyre2 model, while the Alphafold model placed the cleavage site within an α-helix.

A

>AtFbKr

MKRLPLHLLDEILFNLDPKSLGKMRCTNKSINTHISDDPNFKFEYFSRIGSSLLHISKVGSKFLCFYPYAISRLFKNMT
PLKNLCNILGSCSGLVLLSINGILCVANPLTKKFRFLHYSIWGNETWIGFAVDQIDRATQRFKIVFISEQLEVSNPYET
TYQFRINTGESWSLSKTTITCRASNLKKGKNSKPVYVNGDLHWLRKDGSIVAFNPETEKARLIQSQFNRKPGKLLLCTG
DNRLTLISATDAVISVYALETDGQWILVRWIKNEVVHQSLPLLYWNVQAYDGKCLLVRMMSLVGSVIHRYDLRANKWRV
LGSIPTWCDADRDFFLFKPSWSSVIGLLDQEHVHVLMPMPMPMPMPMPMHMHMHMPMPMAMPMPMPIAMAMPMPM
PMPMPMPMTKTETETVTRSEVISSVMAIMGLVNRTLSFIN

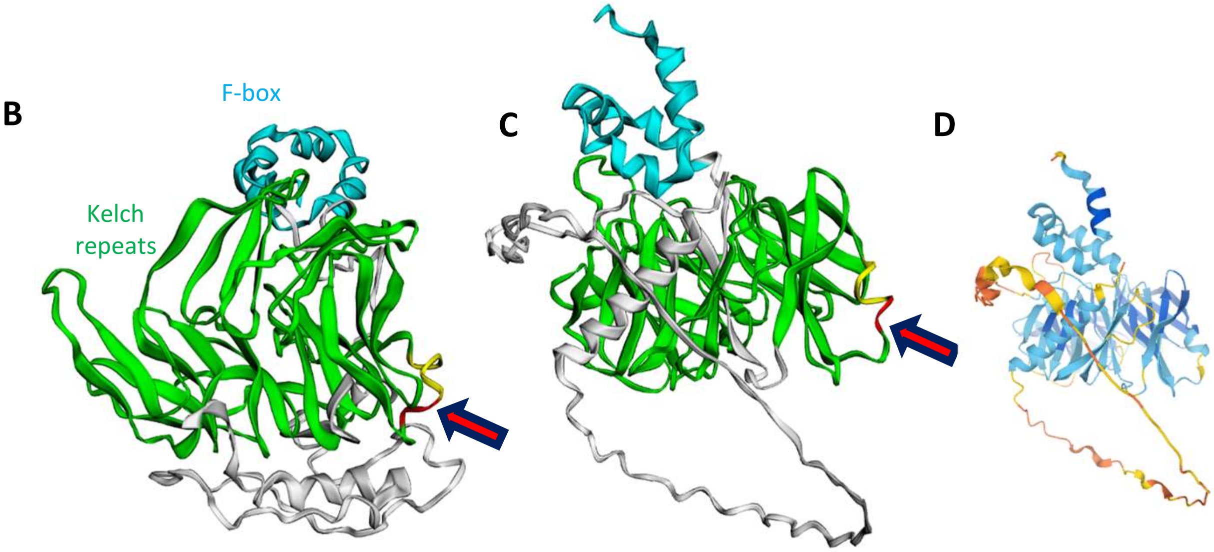

Figure S9. Structural models of the AtFbKr protein **(A)** Sequence of AtFbKr. Predicted F-box and Kelch repeats are underlined in blue and green, respectively. Letters highlighted in yellow correspond to the P6 to P1' positions of the predicted cleavage site with the P1 and P1' positions underlined in red. (**B-D**) Structural models of AtFbKr predicted using Phyre2 (**B**, see Materials and Methods) or Alphafold (**C**, PDB file of the model available at https://www.alphafold.ebi.ac.uk/). For both models, the color scheme is as follows: Kelch repeats in green, F-box in blue, P6 to P2 positions of the predicted cleavage site in yellow and P1 to P1' position of the cleavage site in red. The position of the cleavage site is highlighted with the red arrow. The degree of confidence in the Phyre 2 model **(B)** varied with the domains of the protein. The predicted F-box and kelch repeat domains (highlighted in blue and green, respectively) were modeled with a very high degree of confidence (99.4%) based in part on the solved structure of f-box/wd repeat protein 7 within the skp1-fbw7-cyclinedegc complex (pdb: 2OVQ). Other regions of the protein were modeled *ab silico* with a low degree of confidence. The degree of confidence in the Alphafold model **(C)** also varied with the region of the protein, as shown in **(D)** and using the following color scheme: dark blue (more than 90 %), light blue (70-90 %), yellow (50-70%) and orange (less than 50%). In both models, the cleavage site is located at the base of the highly structured kelch-repeat domain.

A

>PpRHLP

<u>MTVPKTVKDIQSLTGRVAALTRFISKATDRCAPFFKALKGTKRNITWTAECDTAFSELKEYMGRAPLLSTPEH</u>
<u>GDIHVIYLSISASAVSSVLIRSKDNAEHPVHYVSKALQDAEVRYPDIEKLAFALVVSARRLRPYFQAHTIYVL</u>
<u>TNQPLGQVLQNPETSGRLVKWAIELGEFDIHYKPRPAMRGQAVADFLSEFTNPQASAATQLITEPNPPPSQDQ</u>
<u>TPTEGNLDLTQPLWTLFVDGSSNAQGCGAGLVLISLDKVALEYALRFKFQASNNEAEYEALLAGLRLAKEMDA</u>
<u>RQILIFSDSQLVVHQVNQDFTAKDASMTAYLQHARHLLATFHAHSIKQVPRSENSHADALARLASALE</u>QGMGR
HIHIEFLAQPSTQAPLICTIDHSPTWMDPILQFLQNQTLPANPAEARRVRHRSARYLIINGSLYNRGFSLPYL
RFLTPEEGHYVLREIHEGICGNHSGTRSLAHKGNPPRILLAIAPH

B

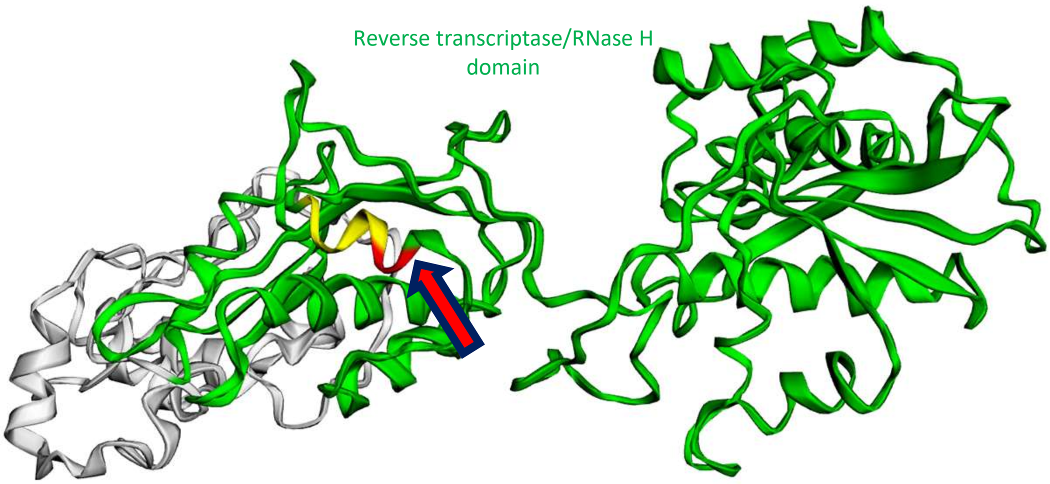

Figure S10. Structural model of the PpRHLP protein (A) Sequence of PpRHLP. A predicted reverse transcriptase/RNase H domain is underlined in green. The letters highlighted in yellow correspond to the P6 to P1′ positions of the predicted cleavage site with the P1 and P1′ positions underlined in red. (**B**) Structural model of PpRHLP predicted using Phyre2 (see Material and Methods). The degree of confidence in the model was high with 87% of residues modelled at 100% degree of confidence based in part on the solved structure of the human immunodeficiency virus-2 reverse transcriptase (pdb: 1MU2). The position of the predicted cleavage site (P6 to P2 position highlighted in yellow; P1 and P1′ positions highlighted in red) is shown with the red arrow. The cleavage site is located within an α–helix in the middle of the highly structured reverse transcriptase/RNAse H domain.

A >AtPRC

MCNCSSSFFCSLPVLNARLVKPNSETCRWRLKRIQHSILNCFWIDSKNSPFLGQFSFIEKPRDNFICCLSSSLSNE
EDVVHQTVGSDSVELPGESDLVRLVGDNDLSITGSRGFKQSTTRSNLVAKQVVSIQSALSLGFISQLWVDTTSWLV
LVVDVKPSLLSGESERFLLTDIVRVGDVVLVDNETVLDTEFKMVGLETLVGYRVVTPGGRNIGKVRGYSFNINSGI
VESLELDSFGVTIIPSSLVSTYRLDVEDIIEVLQDIVVVQEDAASRKQRLTKGLWDAQFDSEYPDVEDLESSSDRR
RRRRNNRSNRKKRDLDDEEWDIFR

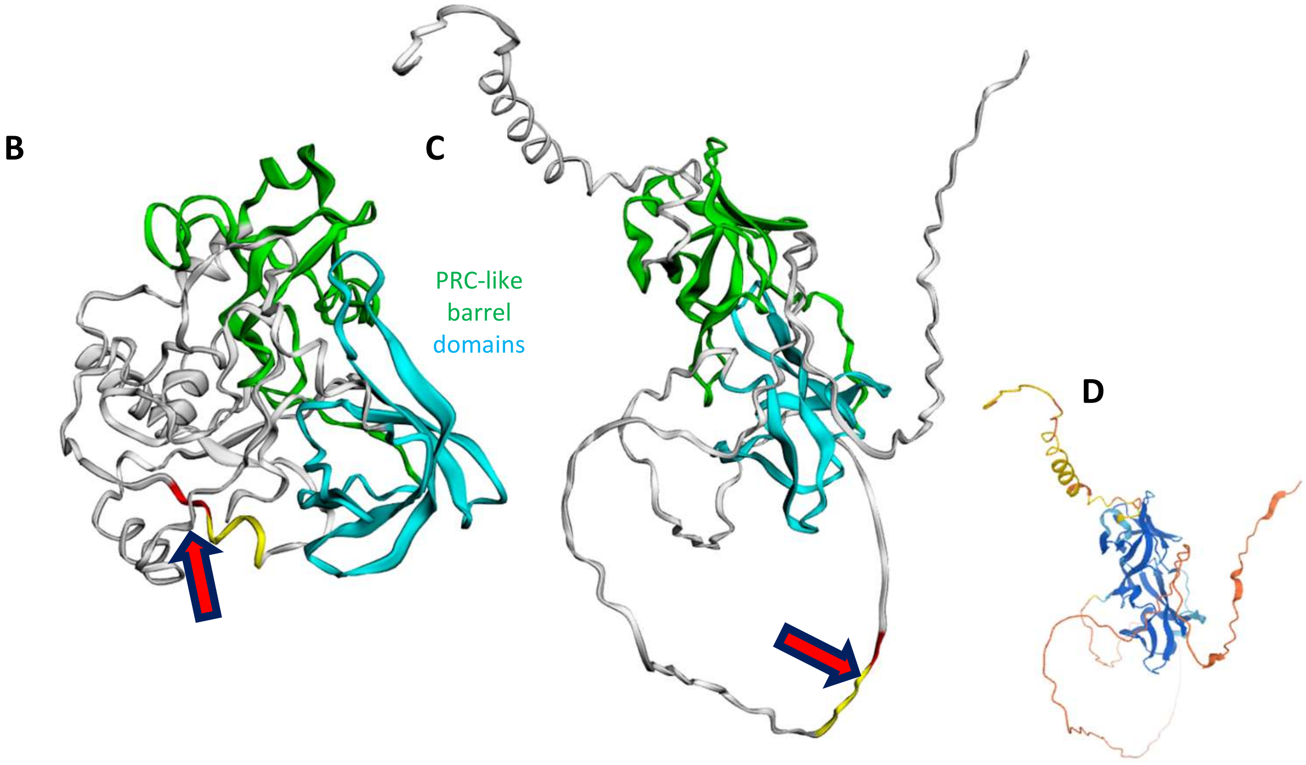

Figure S11. Structural models of the AtPRC protein (A) Sequence of AtPRC. Tandem PRC-like barrel domains are underlined in blue and green. Letters highlighted in yellow correspond to the P6 to P1' positions of the predicted cleavage site with the P1 and P1' positions underlined in red. (**B-D**) Structural models of AtPRC predicted using Phyre2 (**B**, see Materials and Methods) or Alphafold (**C**, PDB file of the model available at https://www.alphafold.ebi.ac.uk/). For both models, the color scheme is as follows: tandem PRC-like barrel domains in blue and green, P6 to P2 positions of the predicted cleavage site in yellow and P1 to P1' position of the cleavage site in red. The position of the cleavage site is highlighted with the red arrow. The degree of confidence in the Phyre 2 model **(B)** varied with the domains of the protein. The predicted tandem PRC-like domains were predicted with a very high degree of confidence (98.1%) based in part on the solved structure of a PRC-barrel domain protein from *Rhodopseudomonas palustris* (pdb: 3HTR). Other regions of the protein (including the region of the predicted cleavage site) were modeled *ab silico* with a low degree of confidence. The degree of confidence in the Alphafold model **(C)** also varied with the region of the protein, as shown in **(D)** and using the following color scheme: dark blue (more than 90 %), light blue (70-90 %), yellow (50-70%) and orange (less than 50%). The position of the cleavage site differed in the two models. In the Phyre2 model, the cleavage site is located within a weakly predicted putative α–helix in an otherwise disorganized region of the protein. In the Alphafold model, the cleavage site is located in a region of the protein predicted to be generally disorganized.
